# Time-Dependent Multimechanistic Antitumor Effects of Olive Oil Phenolics in a Triple-Negative Breast Cancer Mouse Model

**DOI:** 10.3390/nu18172756

**Published:** 2026-08-23

**Authors:** Nikoleta Anna Madelou, Marianna Kapetanou, Katerina Papakonstantinou, Olga Koutsoni, Zacharias Kakazanis, Eleni Melliou, Prokopios Magiatis, Vasilis Zoumbourlis, Efstathios S. Gonos, Haralabia Boleti

**Affiliations:** 1 Hellenic Pasteur Institute, 11521 Athens, Greece; n.madelou@pasteur.gr (N.A.M.); mkapetanou@eie.gr (M.K.); kath.papakonstantinou@gmail.com (K.P.); okoutsoni@pasteur.gr (O.K.); 2 Laboratory of Pharmacognosy and Natural Products Chemistry, Department of Pharmacy, National and Kapodistrian University of Athens, 15771 Athens, Greece; melliou.eleni@gmail.com (E.M.); magiatis@pharm.uoa.gr (P.M.); 3 Institute of Chemical Biology, National Hellenic Research Foundation, 11528 Athens, Greece; zachariaskakazanis@yahoo.gr (Z.K.); vzub@eie.gr (V.Z.); sgonos@eie.gr (E.S.G.)

**Keywords:** olive oil phenolics, oleocanthal, oleuropein aglycone, triple-negative breast cancer (TNBC), MDA-MB-231 xenograft mouse model, antitumor effect, proteasome modulation, protection against protein oxidation, DNA damage, nutraceuticals

## Abstract

**Background/Objectives**: The health-protective properties of olive oil phenolics, including their potential chemopreventive and anticancer effects, have attracted considerable scientific interest. However, their in vivo efficacy and mechanisms of action remain insufficiently understood. Recent advances in extraction and purification technologies have enabled large-scale production of highly purified olive oil phenols and phenolic-rich extracts, facilitating translational research. **Methods**: Herein, the antitumor efficacy of isolated olive oil phenols and phenolic-rich formulations was investigated in an MDA-MB-231 triple-negative breast cancer (TNBC) xenograft model. **Results:** Intraperitoneal administration of oleocanthal (OLC), oleuropein aglycone (OleA) or their combination reduced endpoint tumor burden, with OLC exhibiting the most pronounced effect. Oral administration of total olive oil phenolics (OOPs) achieved comparable efficacy. Pre-treatment initiated before tumor cell implantation conferred the greatest protection, consistent with a prophylactic mode of action. In contrast, delayed intervention displayed diminished or no antitumor benefit. Phenolic-rich extra virgin olive oil likewise showed an inhibition trend in tumor progression. Mechanistically, OOPs attenuated plasma protein oxidation, modulated proteasome mediated proteolysis, and reduced γH2AX levels in vivo. Furthermore, OOPs negatively affected the MDA-MB-231 cell migration in a concentration-dependent manner in vitro. **Conclusions**: Collectively, these findings are consistent with antitumor activities of olive oil phenolics via multiple mechanisms and support their further investigation as prophylactic agents in TNBC and as nutraceuticals.

## 1. Introduction

Breast cancer is one of the most frequently diagnosed malignant solid tumors and remains one of the leading causes of cancer-related mortality among women worldwide. In 2022 alone, approximately 2.32 million new cases were diagnosed, resulting in over 665,000 deaths globally [1,2,3]. Despite treatment advances, including endocrine therapies and targeted agents, the multifactorial and heterogeneous nature of breast cancer continues to highlight the need for novel, well-tolerated therapeutic strategies that target multiple cellular pathways.

Breast cancer is clinically classified according to the positivity of estrogen (ER) and progesterone (PR) receptors, as well as human epidermal growth factor receptor 2 (HER2) gene amplification. This classification defines three major clinical subtypes of HER2 status into hormone receptor-positive, HER2-positive, and triple-negative breast cancer. Further molecular subtyping refines these classifications and guides prognosis and treatment decisions [4,5,6]. Triple-negative breast cancer (TNBC) accounts for approximately 15–20% of cases and is associated with aggressive clinical behavior, high recurrence rates, and limited targeted treatment options [7,8]. Although recent therapeutic advances have expanded treatment strategies, chemotherapy remains the cornerstone of TNBC management [1,9,10,11].

Epidemiological evidence suggests a lower breast cancer incidence in Mediterranean populations compared to other regions [12,13,14,15,16,17,18], as well as in populations adhering to the Mediterranean diet (MD), one of the dietary patterns supported by the greatest accumulated scientific evidence for its beneficial effects on human health [19,20]. Extra virgin olive oil (EVOO), a principal component of the MD, has been associated with protective effects against cancer. These benefits are mainly attributed to its phenolic content, including phenolic alcohols such as hydroxytyrosol and tyrosol, and to secoiridoids, such as OLC, oleacein (OLE), OleA and ligstroside aglycone (LigA) (along with the glycosidic derivatives of the latter two) [21,22,23].

Previous in vivo studies indicate that olive oil phenolic compounds might serve as adjuvant agents in cancer therapy, by enhancing the efficacy of certain chemotherapeutic drugs, with some limited evidence also suggesting a potential chemopreventive role [22,24,25,26,27,28,29,30,31,32]. Although hydroxytyrosol has been the main focus of most breast-cancer-related research [33,34,35,36,37], OLC, one of the most bioactive secoiridoids, has attracted increasing attention due to its potent antiproliferative and cytotoxic effects across various cancer cell lines, as well as for its anti-inflammatory and anticarcinogenic properties [23,24,26,28,29,31,38,39,40,41,42,43,44,45].

Human tumor xenografts in immunodeficient mice remain one of the most widely used pre-clinical models for evaluating anticancer efficacy [46,47,48]. Xenografts can be derived from cultured human cancer cell lines (cell-derived xenografts, CDXs) or patient tumors (patient-derived xenografts, PDXs), typically implanted subcutaneously [49,50,51]. These models enable assessment of tumor growth, therapeutic response, and mechanistic endpoints under controlled in vivo conditions, providing an important bridge between in vitro findings and clinical translation [46].

Our recent ex vivo studies [42,43] demonstrated that olive oil phenolic compounds, including OLC, OLE, OleA and LigA, as well as their combinations and total phenolic extracts, exhibit antiproliferative and cytotoxic effects across multiple cancer cell lines, including the MDA-MB-231 TNBC model. However, direct in vivo comparisons between isolated phenolics and phenolic-rich formulations, as well as the impact of treatment timing on antitumor efficacy, remain limited. This study addresses this knowledge gap by focusing on the administration route, treatment timing and the underlying molecular mechanisms for their prophylactic and oncostatic effects on CB17-SCID mice bearing MDA-MB-231 TNBC xenografts.

## 2. Materials and Methods

### 2.1. Chemicals and Reagents

Isopropanol, dimethyl sulfoxide (DMSO), ethyl acetate, polyethylene glycol 400 (PEG 400), paraformaldehyde (PFA), ammonium chloride (NH_4_Cl) and Hoechst 33342 were acquired from Fisher Scientific (Schwerte, Germany). Deuterated chloroform (CDCl_3_) and 3-(4,5-dimethylthiazol-2-yl)-2,5-diphenyl-2H-tetrazolium bromide (MTT) were obtained from Sigma-Aldrich (Darmstadt, Germany). Fetal bovine serum (FBS), Dulbecco’s modified Eagle medium (DMEM), and Dulbecco’s phosphate-buffered saline (DPBS) were purchased from Capricorn Scientific (Ebsdorfergrund, Germany). Trypsin–EDTA 1× in PBS without calcium, magnesium, or phenol red and penicillin–streptomycin solution (100×; 10,000 U/mL penicillin and 10,000 μg/mL streptomycin) were obtained from Biosera (Cholet, France) and CF^®^633 Phalloidin from Biotium Inc. (Fremont, CA, USA). Rabbit IgG conjugated to Alexa Fluor 647 (ab150079), DAPI-containing antifade mounting medium (ab188804), a Phospho-gamma H2A.X (Ser139) ELISA Kit (ab279816) and a Protein Carbonyl ELISA Kit (ab238536) were from Abcam (Cambridge, UK). The fluorogenic substrate LLVY-AMC (BML-P802) and the MG132 (BML-Pl102proteosome inhibitor were purchased from Enzo Life Sciences (Farmingdale, NY, USA). Mini-PROTEAN TGX Stain-Free™ gels and Bradford assay reagents were from Bio-Rad Laboratories (Hercules, CA, USA).

### 2.2. Extraction of Phenolic Compounds from Extra Virgin Olive Oil

The phenolic extract was isolated using a selective extraction procedure designed to efficiently separate OOPs from the lipid matrix using PEG 400 as the initial extraction medium. The obtained extract, containing 5–10% (*w*/*w*) phenolic compounds, consisted primarily of the dialdehydic secoiridoid OLC along with OLE, OleA and LigA, as well as minor amounts of tyrosol and hydroxytyrosol. For quantitative analysis, aliquots of the extract were dissolved in deuterated chloroform (CDCl_3_) prior to NMR analysis.

To further enrich the phenolic fraction, the crude extract was passed through a column, packed with macroporous food-grade absorption resin, and subsequently eluted with isopropanol. The organic fraction was collected and evaporated under reduced pressure to yield a concentrated phenolic residue. The residue was then reconstituted in PEG 400 to a final concentration of 40% (*w*/*w*), forming a stable phenolic-rich formulation suitable for biological evaluation. This step ensured complete solubilization of the secoiridoids while maintaining their chemical stability.

The enriched OOP formulation contained 40% (*w*/*w*) total phenolics, as determined by quantitative ^1^H NMR. Accordingly, an OOP dose corresponding to 5 mg/kg total phenols delivered 2.7 mg/kg OLC, 1.3 mg/kg OLE, 0.27 mg/kg OleA, 0.64 mg/kg LigA, and 0.05 mg/kg each of hydroxytyrosol and tyrosol. The complete quantitative ^1^H NMR composition of the extract is presented in Supplementary Table S1, and the corresponding ^1^H NMR spectrum is provided in Supplementary Figure S1.

The EVOO was kindly provided by the producers of PAMAKO olive oil (https://www.pamako.gr/product-range/, accessed on 21 June 2026). All treatments were conducted using EVOO from newly opened bottles, which were stored in the dark at room temperature (RT) and used within one month of opening.

### 2.3. Isolation of (−) Oleocanthal and Oleuropein Aglycone

OLC and OleA were isolated in Dr. Prokopios Magiatis’ laboratory (National and Kapodistrian University of Athens, Department of Pharmacy) from the enriched extracts described above, using chromatographic purification.

Briefly, the enriched extracts were subjected to liquid–liquid extraction with ethyl acetate (twice at RT). Water was added to the aqueous phase during each extraction step to ensure complete removal of PEG 400 from the organic layer. The combined organic fractions were filtered and concentrated under reduced pressure at 40 °C to yield a dark brown residue.

Purification was performed using reverse-phase medium-pressure chromatography (RP-MPLC) on C_18_-reversed phase silica gel using a modular BÜCHI Sepacore system: C-615 Pump Manager and C-601 pumps (BÜCHI Labortechnik AG, Flawil, Switzerland). The column dimensions were 2.5 × 50 cm. Elution was carried out using a solvent system of H_2_O (A) and isopropanol (B) with a stepwise gradient with progressively increasing proportions of isopropanol. Fractions containing pure compounds were identified based on UV detection, pooled and concentrated. OLC eluted at an H_2_O:isopropanol composition of 90:10 (*v*/*v*) (fractions 6–22), whereas OleA eluted at 86:14 (*v*/*v*) (fractions 91–100). The ^1^H NMR spectra of the isolated oleocanthal and oleuropein aglycone are provided in Supplementary Figures S2 and S3, respectively.

The identities and purities (≥97%) of the two isolated compounds were confirmed by ^1^H qNMR analysis, as described in Section 2.5. Purified samples were stored in amber glass vials and kept frozen until use.

### 2.4. Preparation of EVOO and Dephenolized Olive Oil

Dephenolized EVOO (dEVOO) was prepared from the same EVOO batch used for animal administration in order to obtain a control with a comparable lipid matrix but with no secoiridoid content. Briefly, EVOO (with phenolic content of similar proportions of phenols to the total phenolic extract in Section 2.2) was subjected to repeated liquid–liquid extraction, four times, with water at 40 °C to efficiently remove mildly water-soluble phenolic constituents.

Following the final extraction, the oil phase was analyzed by ^1^H NMR spectroscopy to confirm the absence of secoiridoid signals and preservation of the lipid profile. The resulting dEVOO served as the control oil in xenograft experiments, allowing comparison with phenolic-rich EVOO while maintaining a similar non-phenolic oil matrix.

### 2.5. NMR Spectral Analysis of Isolated Compounds, OOPs and EVOO

The concentrated extract, OLC, OleA, EVOO and dEVOO were analyzed by quantitative nuclear magnetic resonance (qNMR) spectroscopy. ^1^H NMR spectra were recorded at 400 MHz using an NMR spectrometer (Bruker Avance DRX400 (Bruker, Billerica, MA, USA). All one-dimensional ^1^H-NMR spectra were acquired using a standard 90° excitation pulse (pulse width: 10 μs) with a pre-scan delay of 6.5 s. Measurements were performed at 298 K, typically acquiring 50 scans of 32K data points over a spectral width of 0–16 ppm (5263.18 Hz), with a relaxation delay of 10 s, an acquisition time of 3.11 s and FID resolution of 0.32 Hz. Prior to Fourier transformation (FT), an exponential weighting factor corresponding to a line broadening of 0.3 Hz was applied. Phase adjustment and baseline correction were performed manually using Topspin version 4.07. Signal integration was performed manually using the same resonance region of each peak for all spectra [52]. The ^1^H NMR purity of studied compounds (a total of the two diastereomers in the case of OleA) was >97%. The identity of both compounds was conclusively defined by comparison with literature data [52,53,54,55,56]. Syringaldehyde (98% purity) was used as internal standard (IS), which was purchased from Sigma-Aldrich (Steinheim, Germany). IS solution was prepared in acetonitrile at a concentration of 0.5 mg/mL and kept in a refrigerator. Prior to use the IS solution was left to reach room temperature. OleA and LigA were quantified by integrating doublets at 9.51 and 9.49 ppm, respectively. Oleocanthal and oleacein were measured at 9.23 and 9.19 ppm, respectively, as previously described [53].

### 2.6. In Vivo MDA-MB-231 Xenograft Murine Model and Animal Treatments

The MDA-MB-231/CB17-SCID xenograft model was chosen because it represents one of the most widely established and validated models for investigating the biology and therapeutic response of aggressive TNBC. MDA-MB-231 cells are a well-characterized human breast cancer cell line exhibiting the defining features of TNBC (ER−/PR−/HER2−), a mesenchymal-like phenotype, high invasiveness, and metastatic potential. They have been extensively used in orthotopic and subcutaneous xenograft studies to evaluate tumor growth, invasion, metastasis, and responses to novel therapeutic agents. CB17-SCID (cr-Prkdcscid/scid/Rj) mice provide a permissive host for human tumor engraftment due to the absence of functional T and B lymphocytes while retaining many aspects of the stromal microenvironment. Consequently, they represent a standard platform for human breast cancer xenografts, including MDA-MB-231 tumors [57].

#### 2.6.1. Animals and Ethics Statements

Adult female CB17-SCID (cr-Prkdcscid/scid/Rj) mice, 6–8 weeks old, weighing 15–20 g, were purchased from Charles River Laboratories (Italy, Europe). Animals were maintained in specific pathogen-free conditions at the approved establishments of the Animal Lab Facility of the Institute of Chemical Biology of the National Hellenic Research Foundation (registered codes EL25BIOsup032, EL25BIObr031, EL25BIOexp033). Mice were housed at 22 ± 2 °C, 40–70% relative humidity, under a 12 h light/12 h dark cycle and were given a unique identification number according to the facility’s tail-marking procedure (National Research Foundation’s Experimental Animal Unit). A non-toxic laboratory marker was used to apply the identification code on the distal portion of the tail. Marks were renewed as needed to maintain clarity throughout the experimental period. All procedures complied with PD/56/2013 and European Directive 2010/63/EU for the ethical use of laboratory animals and were conducted in accordance with the PREPARE and ARRIVE guidelines and the principles of the 3Rs. Due to the constraints imposed by the 3Rs principles (Replacement, Reduction, Refinement) and the practical demands of in vivo xenograft work, group sizes of *n* = 4–5 animals were used.

All protocols were approved by the National Research Institute’s Bioethics committee and were performed under the licensed protocol with permission numbers 356688 and 1380634 (dates of approval; 3 March and 10 November 2023, respectively) by the Official Veterinary Authorities of Attica Prefecture.

#### 2.6.2. Tumor Generation

To reduce animal use in accordance with the 3Rs principles, a bilateral orthotopic implantation model was employed, whereby two tumors (one in each mammary fat pad) were established per mouse. This approach generated two tumor specimens per animal for downstream mechanistic assays, enabling the use of only 4–5 animals per group, while providing an adequate number of tumor samples for these assays. Consequently, the biological unit (the individual tumor) and the experimental unit (the mouse, i.e., the smallest unit that could be independently allocated to a treatment) were distinct in this study design [58]. Because the two tumors within a single mouse share systemic factors, including immune status, pharmacokinetics, and host metabolism, they cannot be considered statistically independent. Accordingly, the mouse, rather than the individual tumor, was retained as the experimental unit for statistical inference on the primary efficacy endpoint (tumor burden), consistent with established guidance on avoiding pseudoreplication in pre-clinical research [58,59,60,61].

Animals were randomly allocated to experimental groups before treatment initiation; no formal computer-generated randomization sequence was used. Investigators responsible for group allocation, treatment administration, animal monitoring, and data analysis were aware of treatment assignments.

MDA-MB-231 human breast cancer cells were cultured in T-75 tissue culture flasks (Sarstedt, Nümbrecht, Germany) until the cell monolayer reached approximately 90% confluency. Cells were detached using 0.05% (*w*/*v*) trypsin–EDTA (Biosera, France) for 5 min at 37 °C and resuspended in sterile PBS. A total of 1.5 × 10^6^ cells in 100 μL PBS were injected bilaterally into the mammary fat pads of each mouse to establish orthotopic tumor implantation and a total of 3 × 10^6^ cells per mouse. Mice were randomly assigned to treatment groups. Body weight and general health status were monitored throughout each study to assess general health and treatment tolerability.

#### 2.6.3. Intraperitoneal Administration of Purified Secoiridoids

In the experiment evaluating the antitumor effects of OLC and OleA, mice received intraperitoneal injections of the purified secoiridoids (OLC and OleA), their combination, or DMSO in saline as a vehicle control. All compounds were freshly prepared in 0.9% *w*/*v* saline and administered in a total volume of 100 μL. Treatment was initiated five days after tumor cell implantation and continued three times per week until the experimental endpoint. Animals were euthanized 24 h after the final injection. The OLC dose (5 mg/kg) was selected based on the protocol by Siddique et al., 2019 [44]. The OleA dose (12.5 mg/kg) was determined according to its relative in vitro potency, using a 2.5-fold adjustment based on the previously determined IC_50_ values in our in vitro studies [43]. In the combination group, each compound was administered at half of its corresponding monotherapy dose in order to examine whether co-administration of OLC and OleA doses reduced by half could retain antitumor activity and confirm in vivo the synergistic effect of the two secoiridoids detected in vitro [42].

#### 2.6.4. Oral Administration of Olive Oil Phenolic Extract

The concentrated OOP preparation described in Section 2.2 was diluted in 0.9% (*w*/*v*) saline to deliver the desired dose in a gavage volume of 100 μL per mouse. The final administered solution contained 0.8% (*w*/*v*) PEG 400. The phenolic fraction consisted primarily of OLC and OLE, together with lower amounts of OleA, LigA, hydroxytyrosol and tyrosol. Three independent studies were performed.

In the first study (Section 3.2), mice received OOPs at a dose of 5 mg/kg total phenolic content or the corresponding vehicle (0.9% (*w*/*w*) PEG 400 in saline). Treatment was administered by oral gavage 5 days after MDA-MB-231 cell implantation and was continued three times per week until sacrifice on day 40 after implantation.

In the second study (Section 3.3), the impact of treatment timing was evaluated by initiating OOP administration at one of three time points: 7 days before tumor implantation (OOPs −7 d), 5 days after implantation (OOPs +5 d), or 20 days after implantation (OOPs +20 d). Mice received OOPs at 5 mg/kg three times per week until day 40 after implantation. Food was withheld for 30 min to facilitate optimal absorption. Mice were euthanized 24 h after the final administration.

In the third study (Section 3.3), the prophylactic pre-treatment protocol (OOPs −7 d) was independently repeated using only OOP-treated and vehicle control groups in order to assess reproducibility. For analyses involving the prophylactic treatment group, data from the two independent experiments were pooled to increase statistical power.

#### 2.6.5. Oral Administration of EVOO and dEVOO

Mice received 100 μL of EVOO by oral gavage three times per week. The administered volume corresponded to a total phenolic content of 5 mg/kg, matching the phenolic dose and phenolic profile delivered to the OOP-treated group. Treatment was initiated 7 days prior to MDA-MB-231 cell implantation and continued until day 40 after implantation. A control group received 100 μL of dEVOO according to the same schedule. Both oils were administered neat, without dilution or emulsification. Food was withheld for 30 min after each administration to facilitate optimal absorption. Mice were euthanized 24 h after the final dose.

#### 2.6.6. Sample Collection and Euthanasia

At the experimental endpoints (i.e., 35 and 40 days post implantation), mice were anesthetized using an isoflurane vaporizer system (RWD, Shenzhen, China) connected to an oxygen supply. Anesthesia was induced with 3–4% (*v*/*v*) isoflurane in oxygen (0.8–1.0 L/min) and maintained at 1.5–2.5% (*v*/*v*) via a nose cone until a surgical plane was verified by loss of the pedal withdrawal reflex. Terminal blood collection was performed by cardiac puncture using EDTA-coated tubes. Plasma was obtained by centrifugation at 2000× *g* for 10–15 min at 4 °C, after which the clarified supernatant was aliquoted and stored at −80 °C until analysis. All animals were euthanized under deep isoflurane anesthesia by exsanguination via cardiac puncture, which served as the primary method of euthanasia. Cervical dislocation was subsequently performed as a secondary physical method to ensure death, in accordance with AVMA Guidelines for the Euthanasia of Animals (2020).

Tumors were excised bilaterally with surgical precision, briefly rinsed in ice-cold PBS, blotted dry, weighed, photographed, placed into labeled vials, immediately snap-frozen in liquid nitrogen and stored at −80 °C for subsequent biochemical analyses.

### 2.7. Cell Lines and Culture Conditions

The TNBC MDA-MB-231 cell line (ATCC No: HTB-26), an epithelial human breast adenocarcinoma line, was kindly provided by Dr. P. Lymberi (Immunology Laboratory, Hellenic Pasteur Institute, Athens, Greece). MDA-MB-231 cells are a well-characterized human breast cancer cell line exhibiting the defining features of TNBC (ER−/PR−/HER2−), a mesenchymal-like phenotype, high invasiveness, and metastatic potential. They have been extensively used in orthotopic and subcutaneous xenograft studies to evaluate tumor growth, invasion, metastasis, and responses to novel therapeutic agents.

Cells were used between passages 5 and 15 and were routinely tested for mycoplasma contamination with all tests negative. Cells were grown in 75 cm^2^ tissue culture flasks (Sarstedt, Nümbrecht, Germany) in high-glucose DMEM supplemented with 10% (*v*/*v*) heat-inactivated FBS, penicillin (100 U/mL), and streptomycin (100 μg/mL). Cultures were maintained in a humidified incubator at 37 °C and 5% (*v*/*v*) CO_2_ until the cell monοlayer reached approximately 80–90% confluency, when they were subcultured.

All cell culture procedures were conducted in Class II biological safety cabinets. Solutions and consumables were sterilized by autoclaving and pre-warmed to 37 °C before use.

### 2.8. Cell Viability Assay

Cell viability was assessed using the MTT reduction assay, as previously described [42]. Briefly, MDA-MB-231 cells were seeded into 96-well flat-bottom plates at a density of 9 × 10^3^ cells/well in 200 μL complete DMEM and allowed to adhere for 18 h. Cells were then treated with OOPs at concentrations ranging from 2 to 200 μM for a 72 h period. Subsequently, MTT solution, prepared in fresh culture medium at a final concentration of 0.5 mg/mL, was added to each well and further incubated for 4 h. The resulting dark-blue formazan crystals were dissolved in DMSO, and absorbance was measured at 570 nm using a microplate reader (Dynatech Laboratories MRX, Chantilly, VA, USA). Three independent experiments were performed, each in triplicate. Cell viability was expressed as a percentage of untreated control cells and is presented as mean ± SEM.

### 2.9. Cell Migration Assay

Cell migration was evaluated using a wound healing assay. MDA-MB-231 cells were seeded at a density of 1.2 × 10^5^ cells/well into 24-well flat-bottom plates in 500 μL DMEM supplemented with 5% (*v*/*v*) FBS, penicillin (100 U/mL) and streptomycin (100 μg/mL). After 24 h of incubation at 37 °C and 5% (*v*/*v*) CO_2_ in a humidified atmosphere, the cultures reached approximately 90% confluence, and the medium was replaced with fresh complete DMEM.

A linear scratch was generated across the confluent monolayer using a 20 μL sterile pipette tip, creating a cell-free gap. Detached cells were removed by washing thrice with DPBS. Cells were subsequently treated with OOPs at concentrations corresponding to the IC_50_, IC_50/2_, or IC_50/4_ values and incubated for 24 or 48 h.

The same wound areas were imaged with phase contrast immediately after scratching (0 h), at 24 and at 48 h using a 10× magnification lens and 0.75 digital zoom of a Leica TCS-SP8 (Leica Microsystems GmbH, Wetzlar, Germany) inverted confocal microscope. The mark-and-find function was used to ensure that identical x–y coordinates were captured at each time point.

Images were quantified using ImageJ version 1.54g (NIH, Bethesda, MD, USA) with the Wound Healing Size Tool plugin. The wound region at 0 h was manually defined, and the same region of interest was subsequently applied to the corresponding images at 24 h and 48 h to quantify migration exclusively within the original scratch boundaries. For each condition, two independent experiments were conducted, each with three biological replicates and two fields of view per coverslip. The mean wound area was calculated for each time point. Percentage of wound closure was calculated as:

$$Wound\;closure\;\left(\%\right)=\frac{\left(A_{0h}-A_{t}\right)}{A_{0h}}\times 100$$

where A_0h_ is the original wound area at 0 h and A_t_ is the wound area at the corresponding time point.

### 2.10. Immunofluorescence Staining of MDA-MB-231 Cells

To further validate the effect of OOPs on migratory behavior of MDA-MB-231 cells, immunofluorescence staining was performed, following the same experimental setup described above (Section 2.9). Sterile glass coverslips were placed in 24-well flat bottom plates prior to cell seeding. Cells were treated with OOPs at the IC_50_ concentration determined in Section 2.8 for 12 h to enable visualization of the wound margin while preserving a distinct acellular gap in the control samples.

Following treatment, coverslips were washed twice with DPBS containing 1 mM Ca^2+^ and Mg^2+^ and fixed with 4% (*w*/*v*) PFA for 20 min at RT. Fixation was quenched with 50 mM NH_4_Cl, followed by DPBS washes. F-actin was labeled using phalloidin conjugated to CF^®^633 fluorochrom for 1 h at RT in the dark, and nuclei were stained with Hoechst 33342 (10 μg/mL) for 10 min. After three washes with DPBS, coverslips were mounted on microscope glass slides with Mowiol 4–88 (10% (*w*/*v*) (Merck KGaA, Darmstadt, Germany), 25% (*v*/*v*) glycerol, and 100 mM Tris-HCl (pH 8.5), sealed with nail polish, and stored at 4 °C. Fluorescence imaging of the stained cells inside or around the “scratch” regions was performed on an inverted Leica TCS-SP8 confocal laser-scanning microscope. CF^®^633-phalloidin was excited with a 633 nm laser and emission was collected at 650–710 nm. Hoechst 33342 was imaged with an SP8 confocal microscope using the laser line of 405 nm for excitation. Emission was collected at 415–533 nm. Images were acquired at 1024 × 1024 pixels. Imaging was performed to visualize wound margins and cell positioning (Supplementary Figure S4) and was not used for quantitative analysis of actin organization or cytoskeletal structure.

### 2.11. Measurement of Proteasome Activity

Proteasome chymotrypsin-like (CT-L) activity was measured by an investigator blinded to treatment allocation in tumor lysates obtained from MDA-MB-231 xenograft-bearing mice treated prophylactically with OOPs, vehicle, EVOO or dEVOO as described in Section 2.6.4 and Section 2.6.5. Tumors from three mice per group were homogenized in lysis buffer containing 25 mM Tris/HCl (pH 7.6), 5 mM ATP, 10% (*v*/*v*) glycerol, 20 mM KCl, 1 mM EDTA, 1 mM DTT, 0.2% (*v*/*v*) Nonidet P-40, 10 mM PMSF, and 10 μg/mL aprotinin.

Proteasome CT-L activity was determined by incubating whole cell extract (10 μg of total protein) with the fluorogenic substrate LLVY-AMC (Enzo Life Sciences Inc., Farmingdale, NY, USA) for 30 min at 37 °C. Fluorescence was measured using a Safire II plate spectrofluorometer (TECAN, Männedorf, Switzerland). Specific CT-L activity was calculated by subtracting the residual activity measured in the presence of 20 μM MG132 (Enzo Life Sciences). Protein concentration was measured using the Bradford assay (Bio-Rad Laboratories) with bovine serum albumin (BSA) as the reference standard.

For analysis of proteasomal subunit levels, 20 μg of total protein lysate was mixed with non-reducing Laemmli buffer and resolved by SDS-PAGE on 10% pre-cast Mini-PROTEAN TGX Stain-Free™ gels (Bio-Rad Laboratories, Hercules, CA, USA). Following electrophoresis, total protein load was visualized and quantified using Stain-Free™ technology and the ChemiDoc XRS+ imaging system (Bio-Rad Laboratories Inc., Hercules, CA, USA).

### 2.12. Measurement of Histone γH2A.X Phosphorylation

#### 2.12.1. Quantification of γH2AΧ Levels in Tumor Lysates

The biochemical detection of gamma-H2AX, phosphorylated histone H2A.X on Ser139, a marker of DNA double-strand breaks, was performed by an investigator blinded to treatment allocation in protein extracts from MDA-MB-231 xenograft tumors using the Phospho-gamma H2A.X (Ser139) ELISA Kit (Abcam ab279816, UK), following the manufacturer’s instructions. Equal amounts of total protein from each sample were loaded into the ELISA plate and incubated with the phospho-specific capture and detection antibodies. Following the addition of the substrate solution, colorimetric detection was carried out by measuring the absorbance at 450 nm. γH2A.X levels were determined based on the absorbance values and expressed relative to the vehicle-treated control group.

#### 2.12.2. Immunocytochemical Detection of γH2AΧ in MDA-MB-231 Cells

MDA-MB-231 cells were cultured on glass coverslips and treated for 24 h with isolated olive phenols (OLC, OleA, OLE, LigA) and OOPs at their respective IC_50_ concentrations. Vehicle-treated cells (DMSO) served as controls. Following treatment, cells were fixed with 4% (*w*/*v*) PFA and permeabilized with 0.2% (*v*/*v*) Triton X-100 in PBS. Subsequently, cells were incubated for 1 h at RT with anti-γH2A.X antibody (phospho-Ser139, clone JBW301, 05–636, Millipore, Burlington, MA, USA) at 1:200 dilution, followed by incubation with rabbit IgG conjugated to Alexa Fluor 647 (ab150079, Abcam). Coverslips were mounted onto glass slides using 10 μL of DAPI-containing antifade mounting medium (ab188804, Abcam, UK). Confocal imaging was performed using a Leica TCS-SPE microscope and LAS AF software (Leica Microsystems GmbH, Wetzlar, Germany). Cells displaying more than five γH2A.X positive foci per nucleus were classified as DNA-damage-positive by a single observer blinded to the treatment groups.

### 2.13. Measurement of Oxidized Proteins in Mice Serum

Protein carbonylation was measured using the Protein Carbonyl ELISA Kit (ab238536; Abcam, Cambridge, UK), according to manufacturer’s instructions. All measurements were performed in duplicates. Briefly, serum samples were diluted 100-fold in PBS to a final protein concentration of 10 μg/mL and allowed to absorb onto 96-well plates. Protein carbonyl groups present in oxidized proteins were then derivatized with dinitrophenylhydrazine (DNPH), forming stable DNP hydrazone adducts. The latter were subsequently detected using an anti-DNP antibody. The signal was developed through a horseradish peroxidase (HRP)-conjugated secondary antibody, followed by a chromogenic substrate, allowing spectrophotometric quantification. Absorbance was recorded using a Safire II microplate reader (TECAN, Männedorf, Switzerland) and the levels of carbonylated proteins in the samples were determined from a standard curve generated using BSA standards.

### 2.14. Statistical Analysis

Data are presented as mean ± SD or mean ± SEM, as indicated. Normality and homogeneity of variance were assessed prior to analysis. Comparisons between treatment groups were performed using one-way ANOVA followed by Tukey’s or two-way repeated-measures ANOVA followed by Dunnett’s post hoc test, as appropriate. Differences between two groups were analyzed using an unpaired two-tailed Student’s *t*-test. Effect sizes were calculated using Hedges’ g for in vivo experiments, which is appropriate for small sample sizes [62,63]. Outliers were identified using the ROUT method (Q = 1%) and excluded from analysis where indicated. ROUT analysis was applied only to identify extreme tumor measurements. Outlying tumor measurements were excluded, and the remaining tumor measurement for that mouse was retained. The mouse-level tumor burden was calculated after excluding the outlying tumor measurement. Statistical comparisons were then performed using one observation per mouse (*n* = number of mice). Statistical significance was defined as *p* < 0.05. All analyses were conducted using GraphPad Prism (version 8).

## 3. Results

### 3.1. Effects of Intraperitoneal Administration of OLC and OleA on Breast Tumor Growth

**Figure 1:**
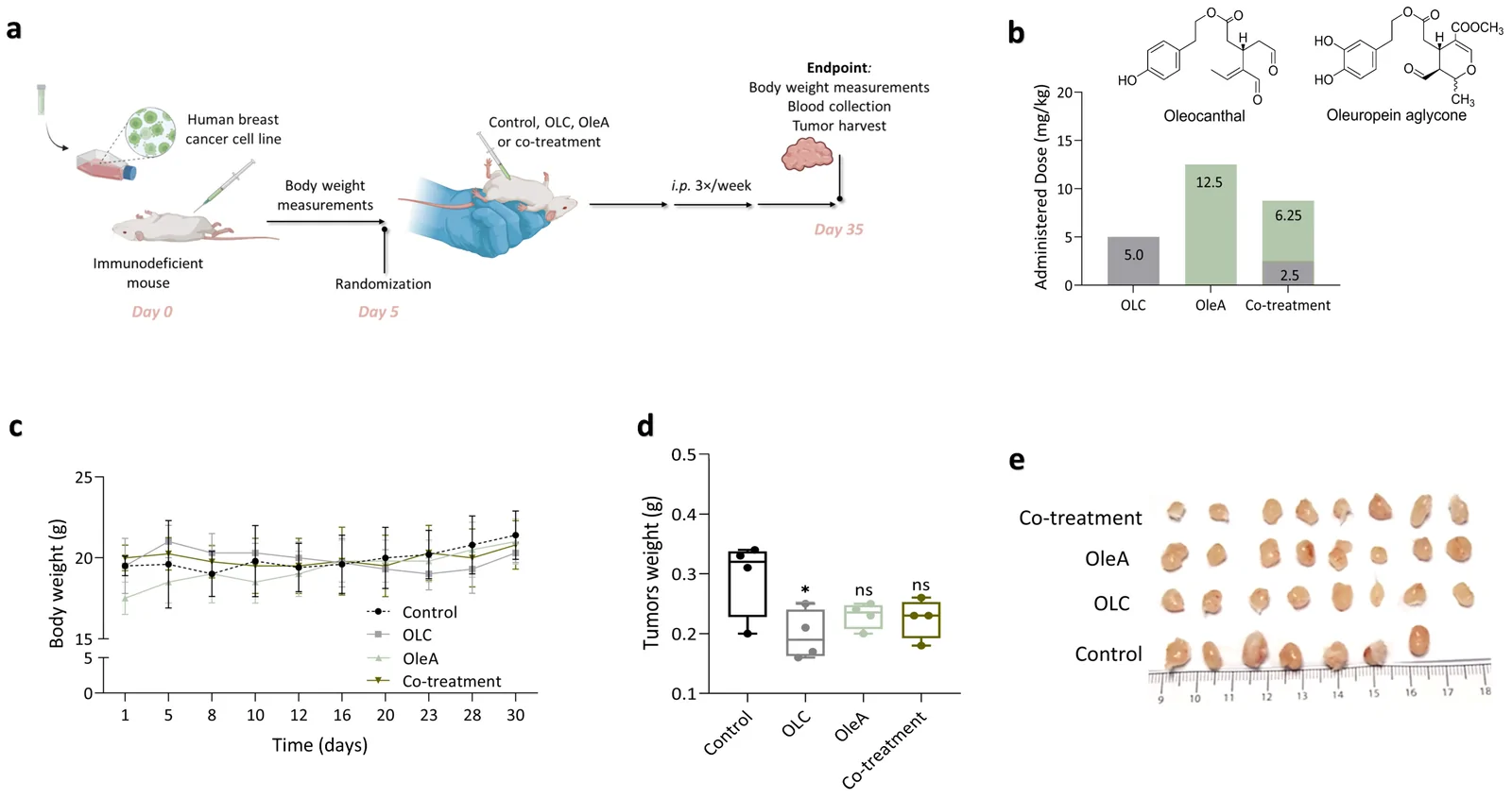
Effect of intraperitoneal administration of OLC, OleA and their combination on tumor growth in MDA-MB-231 xenograft-bearing mice. (**a**) Schematic representation of the experimental design. CB17-SCID mice were injected bilaterally into the mammary fat pads with MDA-MB-231 cells (Day 0) to establish orthotopic xenograft tumors. Five days after implantation of cancer cells (Day 5), mice received intraperitoneal treatments three times per week for 30 days. The experimental groups included OLC (5 mg/kg), OleA (12.5 mg/kg), OLC + OleA co-treatment (2.5 mg/kg and 6.25 mg/kg, respectively) and a vehicle control (DMSO). Mice were euthanized 24 h after the final treatment for tumor and blood collection. (**b**) Chemical structures and administered doses (mg/kg) of individual secoiridoids and their combination. (**c**) Body weight monitoring throughout the study. Data are presented as mean ± SEM. (**d**) Endpoint tumor burden calculated as the sum of the weights of the bilateral tumors for each mouse (total tumor burden). For the single mouse in the control group that developed only one tumor the weight of that tumor was used as the total tumor burden. Box-and-whisker plots show median, interquartile range, and minimum-to-maximum values; each point represents one mouse (*n* = 4 mice per group). Statistical analysis was performed using one-way ANOVA followed by Tukey’s multiple comparisons test. Data are presented as mean ± SD. * *p* < 0.05. (**e**) Representative images of tumors excised from mice of each treatment group at the study endpoint. Abbreviations: OLC, oleocanthal; OleA, oleuropein aglycone.

To evaluate the in vivo antitumor activity of purified olive oil secoiridoids, MDA-MB-231 xenograft-bearing SCID mice were treated intraperitoneally with OLC, OleA, or a combination of both compounds, according to the protocol described in Section 2.6.3 (Figure 1a,b). Treatment with each secoiridoid as well as their combination noticeably reduced final tumor burden compared with the vehicle control group (Figure 1).

Successful tumor establishment was achieved in all animals, with bilateral tumors developing in every mouse. The OLC-treated group exhibited the most pronounced effect on tumor weight, showing a 33.1% reduction (95% CΙ: 0.01576 to 0.1792; *p* = 0.0197; Hedges’ g = 1.57). Due to the small sample size (*n* = 4 mice per group), Hedges’ g was used instead of Cohen’s d for all groups to minimize bias. OleA treatment reduced tumor weight to 0.23 g, corresponding to a 22.0% decrease (95% CΙ: −0.01674 to 0.1467; *p* = 0.1293) compared with the control group and a large effect size as well (Hedges’ g = 1.17), although not statistically significant. Combined treatment with OLC and OleA decreased tumor weight to 0.225 g (23.7% reduction; 95% CΙ: −0.01174 to 0.1517; *p* = 0.0980), corresponding to Hedges’ g = 1.19 (Figure 1d,e).

Although the combination treatment inhibited tumor growth compared with the control group, it was less effective than OLC monotherapy under the conditions tested. However, it should be noted that each secoiridoid was administered at half the dose used in the corresponding monotherapy groups. Therefore, the combination retained marked antitumor activity despite the half dose of both compounds, suggesting that biological efficacy was preserved under these experimental conditions.

Importantly, no significant changes in body weight were observed among groups during the study (Figure 1c) and in the overall well-being of mice, indicating that all treatments were well tolerated.

### 3.2. Effect of Orally Administered OOP Extract on Breast Tumor Growth

To evaluate whether the antitumor activity of secoiridoid compounds could also be achieved through oral administration, MDA-MB-231 xenograft-bearing mice received the OOP extract (5 mg/kg, enriched in OLC), as described in Section 2.6.4.

**Figure 2:**
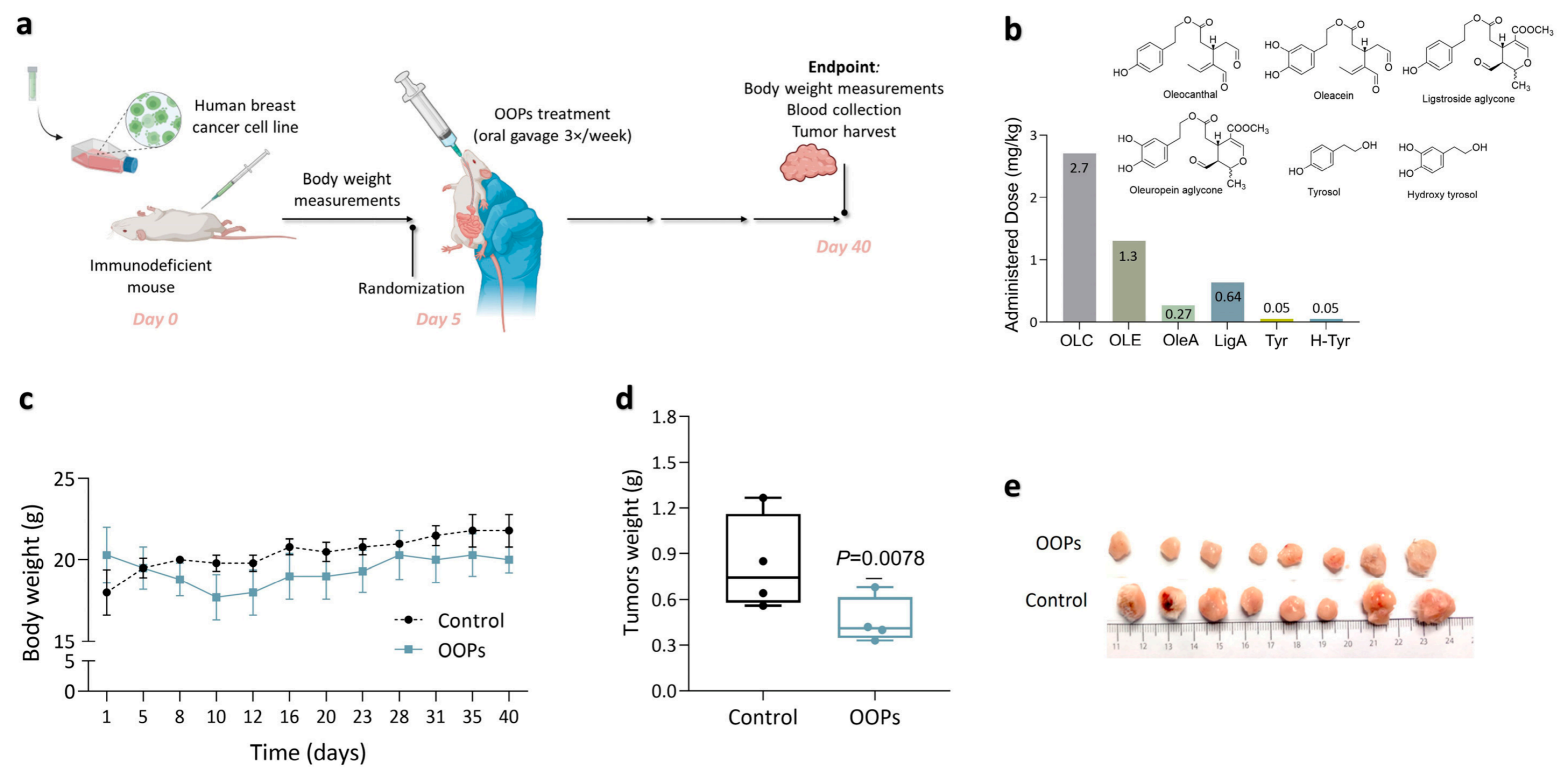
Effect of oral administration of OOPs on tumor growth in MDA-MB-231 xenograft-bearing mice. (**a**) Schematic overview of the experimental design. CB17-SCID mice were injected bilaterally into the mammary fat pads with MDA-MB-231 cells (Day 0) to establish orthotopic xenograft tumors. Five days post implantation of cancer cells (Day 5), mice received orally either OOPs (5 mg/kg body weight) or vehicle control (PEG 400 in saline) three times per week throughout the study. Mice were euthanized 24 h after the final treatment for tumor and blood collection. (**b**) Chemical structures and administered doses (mg/kg body weight) of individual secoiridoids within the OOP mixture. (**c**) Body weight monitoring throughout the study. Data are presented as mean ± SEM. (**d**) Endpoint tumor burden calculated as the sum of the weights of the bilateral tumors for each mouse (total tumor burden). Box-and-whisker plots show median, interquartile range, and minimum-to-maximum values; each point represents one mouse (*n* = 4 mice per group). Statistical analysis was performed using an unpaired two-tailed Student’s *t*-test. Data are presented as mean ± SD. (**e**) Representative images of tumors excised from animals of each group at the study endpoint. Abbreviations: OOPs, total olive oil phenolic extract; OLC, oleocanthal, OleA, oleuropein aglycone; OLE, oleacein; LigA, ligstroside aglycone; Tyr, tyrosol; H-Tyr, hydroxytyrosol.

Successful tumor formation was observed in all animals, with bilateral tumors developing in every mouse. Oral administration of OOPs resulted in a marked reduction in tumor weight compared to the control group. However, although tumor weight was reduced by 44.8%, this difference did not reach statistical significance (95% CI: −0.8011 to 0.05814 g; *p* = 0.078) (Figure 2d,e). The corresponding effect size (Hedges’ g = 1.30) suggests a potentially large treatment effect. However, given the limited sample size, this observation should be interpreted with caution and considered indicative rather than conclusive.

No significant weight loss or toxicity was observed in treated animals (Figure 2c), indicating that oral administration of OOPs was well tolerated under the conditions tested.

### 3.3. Effect of Timing in the Oral Administration of OOP Extract on Breast Tumor Growth

To determine whether the timing of treatment initiation influences the antitumor efficacy of OOPs administered orally, MDA-MB-231 xenograft-bearing mice received OOPs either 7 days prior to tumor cell implantation (−7 d, prophylactic pre-treatment group), 5 days after implantation (+5 d, early-treatment group), or 20 days after implantation (+20 d, late-treatment group).

**Figure 3:**
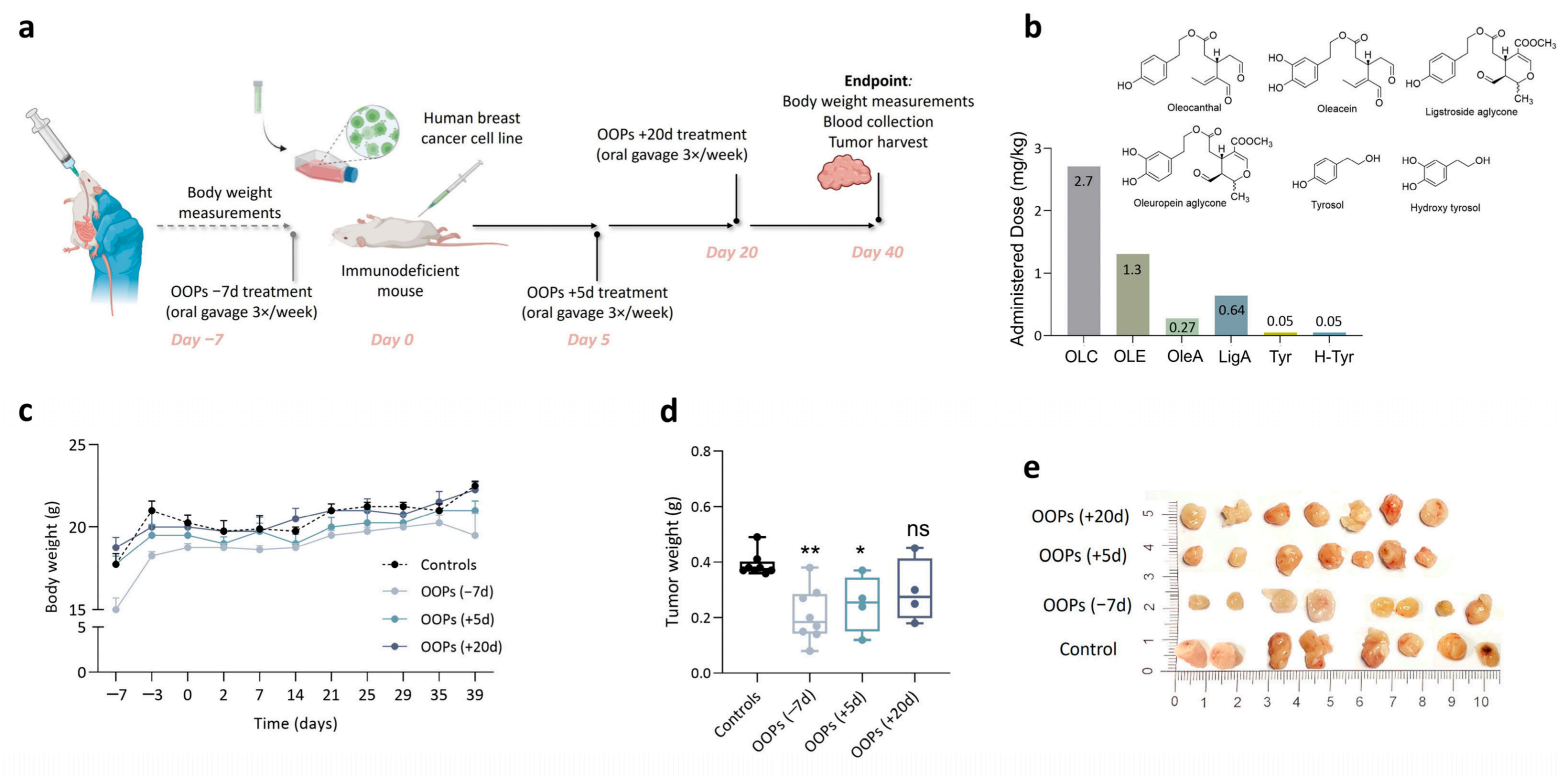
Timing-dependent antitumor effects of orally administered OOPs in an MDA-MB-231 xenograft-bearing mouse model. (**a**) Schematic representation of the experimental design. CB17-SCID mice received oral OOPs at a dose corresponding to 5 mg total secoiridoid content/kg body weight, starting 7 days before (−7 d) and 5 (+5 d) or 20 (+20 d) days after implantation of MDA-MB-231 human TNBC cells (Day 0). Vehicle control animals received PEG 400 in saline. Treatments were administered three times per week throughout the study. Mice were euthanized 24 h after the final dose; tumors and blood samples were subsequently collected. (**b**) Chemical structures and administered doses (mg/kg) of individual secoiridoids within the OOP mixture. (**c**) Body weight monitoring throughout the study. Data are presented as mean ± SEM. (**d**) Endpoint tumor burden calculated as the sum of the weights of the bilateral tumors for each mouse (total tumor burden). Four animals (2 from the prophylactic treatment (−7 d), one from the +5 d early-treatment group and one from the +20 d late-treatment group) had one of the two tumors identified as outliers by the ROUT method (Q = 1%), because of its unusually large weight. These tumors were excluded from final analysis and only the weight of one tumor/mouse was used in the analysis as total tumor burden of those mice. Vehicle-treated control and (−7 d) OOP-treated animals from two independent experiments were pooled together for analysis (*n* = 7–8 mice per group). From the analysis, one animal from the −7 d group that did not develop any tumor was excluded. Box-and-whisker plots show median, interquartile range, and minimum-to-maximum values; each point represents one mouse (*n* = 4–8 mice per treatment group). Statistical analysis was performed using one-way ANOVA followed by Tukey’s multiple comparisons test. Data are presented as means ± SD values. *: *p* < 0.05; **: *p* < 0.01; ns: not significant. (**e**) Representative images of tumors excised from animals of each group at the study endpoint. From the vehicle control and the −7 d groups only the tumors from four animals are shown. Abbreviations: OOPs, total olive oil phenolic extract; OLC, oleocanthal, OleA, oleuropein aglycone; OLE, oleacein; LigA, ligstroside aglycone; Tyr, tyrosol; H-Tyr, hydroxytyrosol.

OOPs (5 mg/kg) were delivered by oral gavage, three times per week, as described in Section 2.6.4. Body weight remained stable throughout the study and no signs of toxicity were observed in any treatment group, indicating good systemic tolerability of the OOP regimen (Figure 3c).

Each mouse carried two tumors except one mouse from the prophylactic pre-treatment group (−7 d) which did not develop any tumor and was excluded from the analysis. Four tumors (one tumor from four different mice) were identified as outliers by ROUT (Q = 1%) analysis. For these mice, the outlying tumor measurement was excluded and the remaining tumor measurement was used for that mouse. The mouse-level tumor burden was calculated after excluding the outlying tumor measurement. The statistical comparisons were then performed using one observation per mouse (*n* = number of mice). The outlier affected only one of the paired tumors while the contralateral tumor showed no abnormal characteristics, so excluding only the aberrant measurement allowed retention of the valid animal-level observation.

Tumor burden was influenced by the timing of treatment initiation (Figure 3). Prophylactic pre-treatment administration of OOPs, beginning 7 days before tumor implantation, produced the greatest antitumor effect, reducing the tumor burden by 46.0% (95% CΙ: 0.07154 to 0.2838 g; *p* = 0.0008) compared with the vehicle control group, with a large effect size (Hedges’ g = 1.9) (Figure 3d,e). When treatment was initiated 5 days after implantation (early-treatment group), the tumor burden was reduced by 35.0% (95% CI: 0.003152 to 0.2747 g; *p* = 0.0439) relative to controls, corresponding to a moderate-to-large effect size (Hedges’ g = 1.46) (Figure 3d,e). In contrast, no detectable inhibition of tumor growth was observed in the +20 d late-treatment group (95% CI: −0.04435 to 0.2272 g, *p* = 0.2559), in which OOP administration was initiated 20 days after tumor cell implantation (late-treatment group) (Figure 3d,e). This finding suggests that delaying treatment initiation may diminish the antitumor efficacy of OOPs once tumors are established. In sensitivity analyses (Supplementary Table S2) including all observations (with or without ROUT-based exclusions), as expected due to the small sample size, statistical significance was affected in all treatment groups, without, however, altering the overall pattern of treatment effects, and the comparisons were consistent across analyses.

Collectively, these findings indicate that the timing of OOP administration critically influences their antitumor efficacy. The most pronounced protective effects were observed when treatment was initiated prior to tumor establishment, supporting a potential prophylactic role for olive oil phenolics in tumorigenesis.

### 3.4. Effect of Phenolic-Rich EVOO on Breast Tumor Growth

To further elucidate the contribution of olive oil secoiridoids to the observed antitumor activity, mice were orally administered either phenolic-rich EVOO or dephenolized EVOO (dEVOO), as described in Section 2.6.5. Treatment was initiated 7 days prior to tumor cell implantation and was continued throughout the study. The administered volume of EVOO (100 μL/mouse) corresponded to a total phenolic dose of 5 mg/kg body weight.

**Figure 4:**
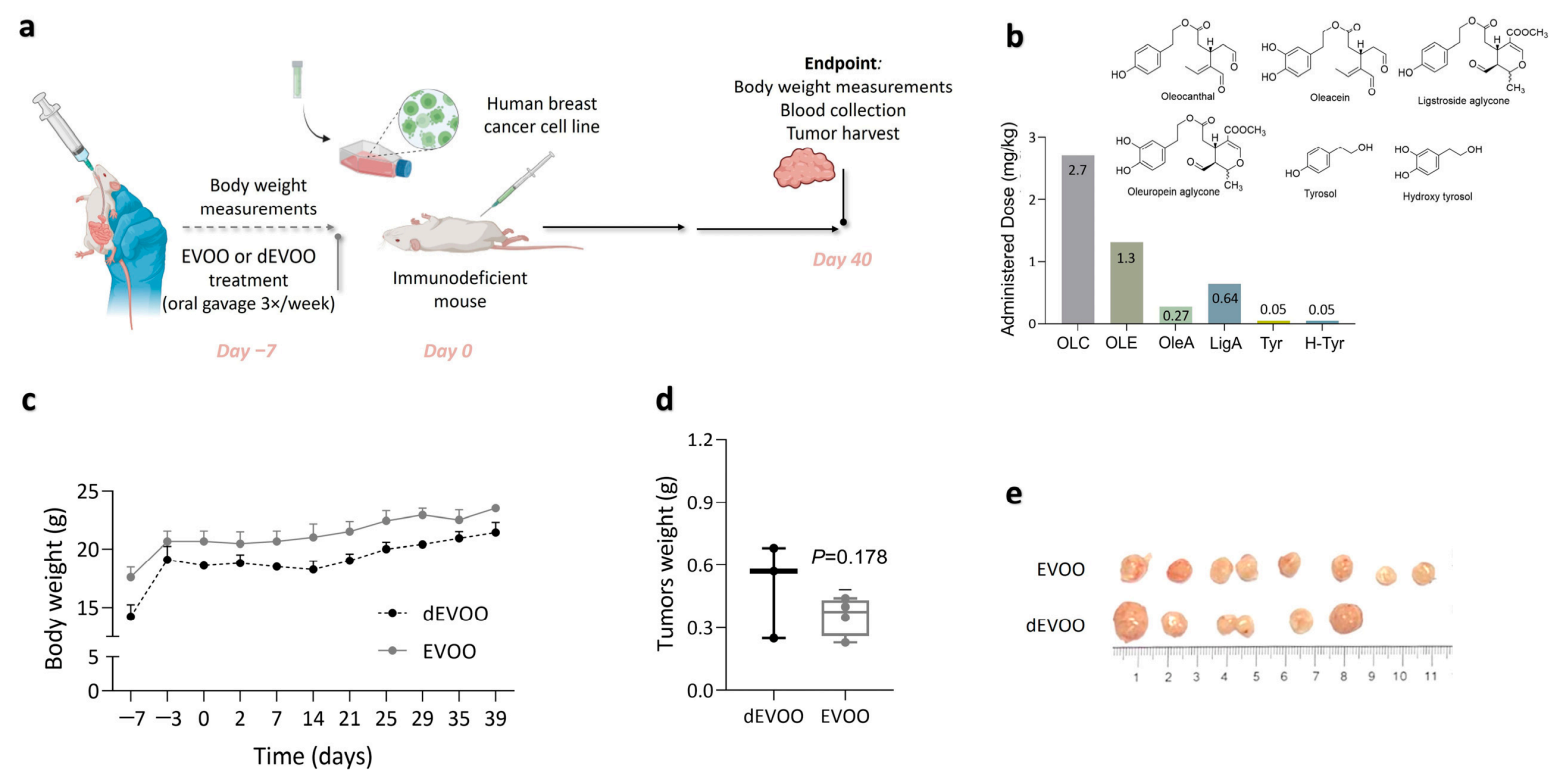
Effect of oral administration of phenolic-rich EVOO on tumor growth in MDA-MB-231 xenograft-bearing mice. (**a**) Schematic representation of the experimental design. CB17-SCID mice received oral EVOO at a dose corresponding to 5 mg total secoiridoid content/kg body weight, starting 7 days before implantation of MDA-MB-231 human TNBC cells (Day 0). Vehicle control animals received dEVOO (negligible secoiridoid content). Treatments were administered three times per week throughout the study. Mice were euthanized 24 h after the final dose; tumors and blood samples were subsequently collected. (**b**) Chemical structures and administered doses (mg/kg body weight) of secoiridoids present in EVOO. (**c**) Body weight monitoring throughout the study. Data are presented as means ± SEM. (**d**) Endpoint tumor burden calculated as the sum of the weights of the bilateral tumors for each mouse (total tumor burden). Box-and-whisker plots show median, interquartile range, and minimum-to-maximum values; each point represents one mouse (*n* = 3–4 mice per group). Tumor formation was observed in all animals, with the exception of one mouse in the dEVOO group, which did not develop any detectable tumor and was excluded from tumor-weight analysis. Statistical analysis was performed using an unpaired two-tailed Student’s *t*-test. Data are presented as mean ± SD (*p* < 0.05 considered significant). (**e**) Representative images of tumors excised from animals of each group at the study endpoint. Abbreviations: OOPs, total olive oil phenolic extract; OLC, oleocanthal, OleA, oleuropein aglycone; OLE, oleacein; LigA, ligstroside aglycone; Tyr, tyrosol; H-Tyr, hydroxytyrosol; EVOO, extra virgin olive oil; dEVOO, dephenolized olive oil.

EVOO administration reduced the sum tumor weight from 0.500 g in the dEVOO group to 0.355 g, corresponding to a 29.0% reduction (Figure 4d,e). Although this difference did not reach statistical significance (95% CΙ: −0.4713 to 0.1148 g; *p* = 0.178), a moderate effect size was observed (Hedges’ g = 0.77), suggesting a potentially meaningful treatment effect. However, given the limited sample size, this result should be considered exploratory and requires confirmation in adequately powered studies.

In addition, and consistent with the results observed in previous experiments, EVOO administration did not cause significant changes in body weight over the course of the experiment (Figure 4c), indicating good tolerability of the treatment.

### 3.5. Effects of Olive Oil Phenolic Compounds on Proteasome Activity in Breast Tumors from a Xenograft Mouse Model

**Figure 5:**
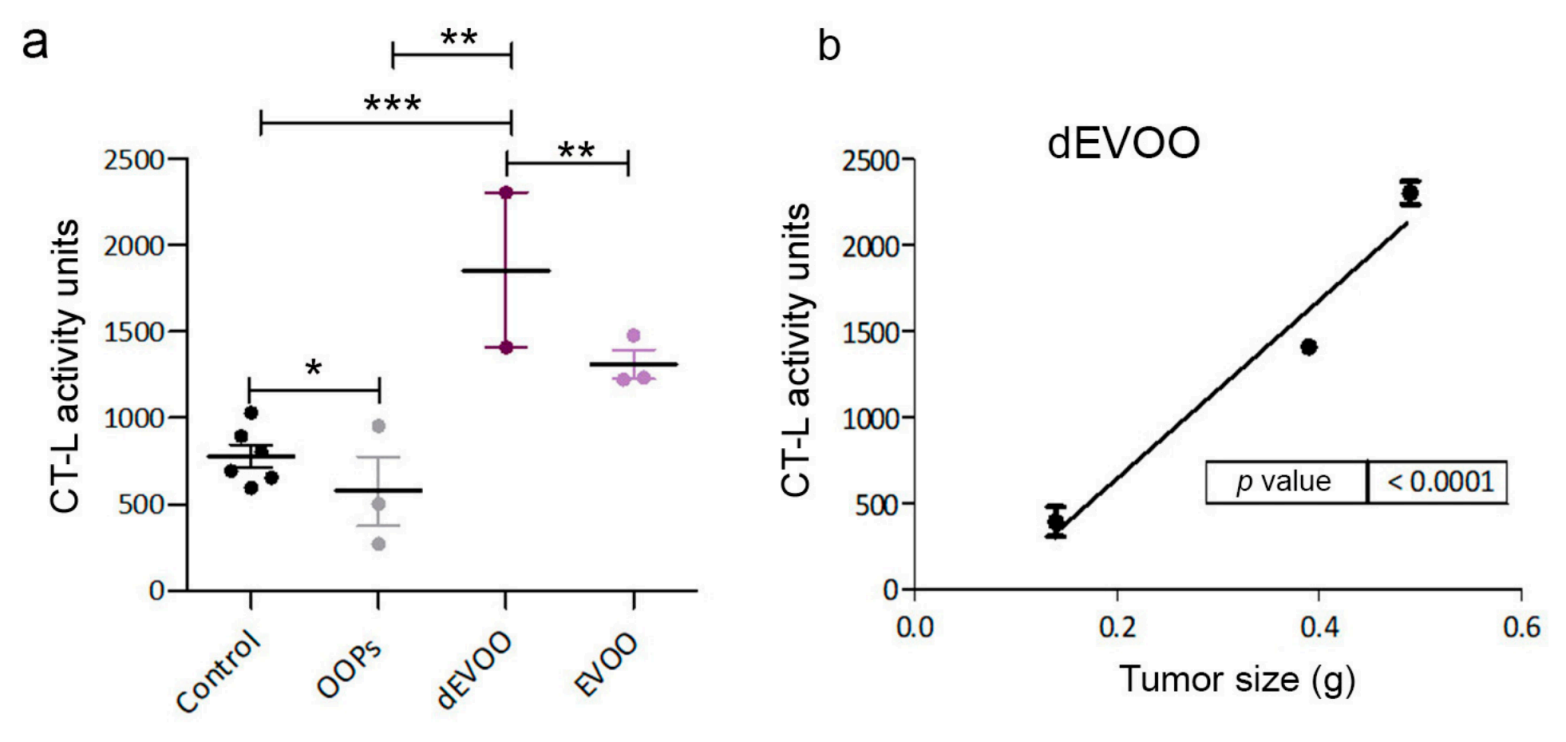
Proteasome activity in xenograft tumors following treatment with olive oil phenolic compounds or phenolic-rich EVOO. (**a**) In vivo analysis of CT-L proteasome activity in tumor lysates from MDA-MB-231 xenograft tumor-bearing mice treated orally with OOPs, dEVOO or phenolic-rich EVOO. The vehicle control group received PEG 400 in saline. Treatment started 7 days before tumor cell implantation. Three tumors were analyzed/group (1 per animal; *n* = 3 mice). In the vehicle control group, animals from the two experiments of prophylactic treatment were analyzed (*n* = 6 mice). Statistical analysis was performed using unpaired two-tailed *t*-tests to compare two groups. Data are presented as mean ± SEM. ***: *p* < 0.001; **: *p* < 0.01; *: *p* < 0.05. (**b**) Correlation between CT-L proteasome activity and tumor size in the dEVOO-treated group: Linear regression analysis indicates a positive association between tumor weight and proteasome activity. Abbreviations: OOPs, total olive oil phenolic extract; EVOO, extra virgin olive oil; dEVOO, dephenolized olive oil.

Proteasome is the primary system responsible for regulated protein degradation and is essential for sustaining the high protein turnover characteristics of malignant cells [64]. Previous studies have shown that olive oil phenolic compounds, including hydroxytyrosol and oleuropein derivatives, can modulate proteasome-related processes—particularly in non-cancerous cells [65]. In this context, we investigated whether a defined phenolic extract (OOPs) derived from EVOO or an EVOO rich in OLC and other secoiridoids can modulate chymotrypsin-like proteasome activity in xenograft-derived tumors in vivo. Proteasome activity was assessed in tumor lysates from mice treated with OOPs or polyphenolic-rich EVOO and compared to the respective control groups (PEG 400 vehicle or dephenolized EVOO). Mice treated with OOPs starting 7 days prior to tumor cell implantation (prophylactic pre-treatment group) exhibited a statistically significant 30% reduction in chymotrypsin-like (CT-L) proteasome activity compared to the vehicle control group (control; *p* = 0.03) (Figure 5a).

Tumors from mice treated with EVOO rich in polyphenols and OLC also exhibited a significant reduction in CT-L proteasome activity compared to those from mice treated with dEVOO (*p* = 0.007). Interestingly, mice treated with dEVOO exhibited significantly higher CT-L proteasome activity than both vehicle control animals receiving PEG 400 (*p* < 0.001) and OOP-treated mice (*p* < 0.001), suggesting that the presence of non-phenolic bioactive compounds in olive oil may enhance proteasomal activity in vivo. Moreover, a significant difference was observed between EVOO- and OOP-treated mice (*p* < 0.01) despite receiving equivalent doses of phenolic compounds for the same length of time. This further suggests that non-phenolic constituents in EVOO may contribute to differences in proteasome-related activity in vivo.

High proteasome activity is essential for tumor progression and it is generally elevated in cancerous tissues compared to normal tissues, a phenomenon that facilitates rapid tumor growth, survival, and metastasis. The specific relationship with tumor size varies by cancer type and stage of development [64,66].

To analyze the large variability observed in the data for CT-L activity in all the groups and particularly in the dEVOO group, the tumor size was correlated to the CT-L activity in the dEVOO-treated mice. Interestingly, a positive correlation was observed (Figure 5b). Smaller tumors exhibited lower CT-L activity, whereas larger tumors displayed higher activity within the same treatment group. This correlation probably contributed to the variability in CT-L activity detected across individual tumors in all mice groups.

Taken together, these findings are consistent with an association between CT-L proteasome activity, treatment composition and tumor burden in vivo, although additional mechanistic studies are required to establish a causal relationship.

### 3.6. OOPs Are Associated with Histone H2A.X Phosphorylation (γH2A.X) Levels in MDA-MB-231 Cells and Xenograft Breast Tumors

To investigate whether OOPs modulate DNA-damage-related signaling, we assessed the phosphorylation of histone H2A.X (γH2A.X), a well-established marker of DNA double-strand breaks. Excessive or unpaired DNA damage can trigger cell cycle arrest, senescence, or apoptosis.

**Figure 6:**
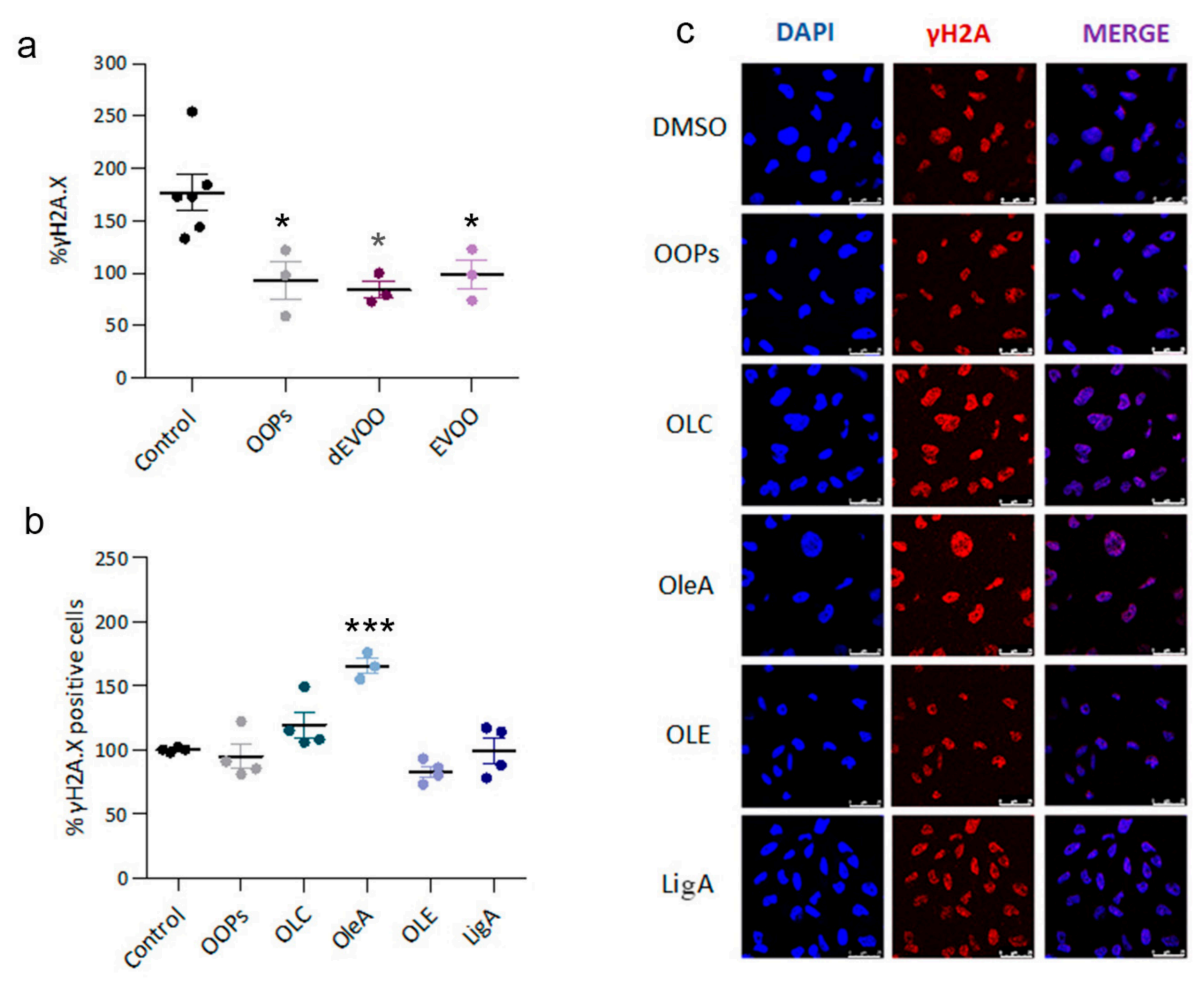
Effects of olive oil phenolic compounds on γH2A.X levels in xenograft tumors and MDA-MB-231 cells. (**a**) γH2A.X levels in tumors isolated from mice treated prophylactically by oral gavage with either vehicle control, OOPs, dEVOO or EVOO. Results are from tumors originating from 3 mice (1 tumor/mouse; *n* = 3 mice/group). (**b**) Quantification of the percentage of γH2AΧ-positive cells. (**c**) Representative images of MDA-MB-231 breast cancer cells after a 24 h incubation with OOPs, OLC, OleA, OLE and LigA at concentrations equal to their respective IC_50_ values [43]. OOPs were used at the IC_50_ value of the OLC content. Intensity signal was increased by 20% for all images for better presentation. γH2A.X was detected with an anti-γH2A.X antibody (and a secondary antirabbit antibody conjugated to Alexa Fluor 647 (red)). DNA was stained with 4′, 6′-diamidino-2-phenylindole (DAPI) (blue). Confocal images were acquired with a 40× apochromat lens using a Leica TCS-SPE microscope (Leica Microsystems) with LAS AF software. Max projections were generated from z-stacks acquired with a step size 0.2 μm. Fluorescence intensity of γH2A.X-positive signal in MDA-MB-231 cells was quantified using Fiji software. More than 100 cells from four randomly selected optical fields were analyzed per condition. The % positive cells in each optical field were plotted per condition (*n* = 4 optical fields). Size bar: 25 μm. Statistical analysis was performed using one-way ANOVA followed by Tukey’s post hoc test. Data are presented as mean ± SEM. Significance relative to control is indicated: ***: *p* < 0.001; *: *p* < 0.05. Abbreviations: OOPs, total olive oil phenolic extract; OLC, oleocanthal, OleA, oleuropein aglycone; OLE, oleacein; LigA, ligstroside aglycone; EVOO, extra virgin olive oil; dEVOO, dephenolized olive oil.

γH2A.X levels were quantified in tumor extracts from xenograft-bearing mice treated with OOPs or polyphenol-rich EVOO and compared with the respective control groups (PEG 400 vehicle or dephenolized extra virgin olive oil, dEVOO). All treatments were administered prophylactically beginning 7 days prior to tumor cell implantation. γH2A.X levels were measured using an ELISA-based assay (Section 2.12). All treatment groups (OOPs, dEVOO and EVOO) showed a significantly lower reduction in the γH2A.X levels compared to the vehicle control (PEG 400) (Figure 6a). Specifically, prophylactic pre-treatment administration of OOPs resulted in an approximately 47% reduction in γH2A.X levels relative to PEG-400-treated mice. Interestingly, the comparable reduction in γH2A.X observed in both the EVOO and dEVOO groups suggests that the biological response cannot be attributed exclusively to the phenolic fraction. These findings raise the possibility that other bioactive constituents of olive oil, in addition to polyphenols, may also contribute to the modulation of γH2A.X-associated DNA damage signaling.

γH2A.X formation was subsequently analyzed in MDA-MB-231 cells following 24 h exposure to OOPs or to individual secoiridoids (OLC, OLE, OleA, LigA) at their respective IC_50_ concentrations [43] using an immunocytochemistry-based assay (Section 2.12). Treatment with OOPs did not significantly alter γH2A.X levels compared to untreated cells (control). OLC induced a modest, non-significant increase in γH2A.X, whereas OLE produced a larger but non-significant decrease. LigA yielded values comparable to controls (Figure 6c). In contrast, OleA induced a pronounced increase in γH2A.X-positive cells (approximately 165% of control), representing the largest effect amongst the tested compounds (Figure 6c).

Overall, γH2A.X levels were significantly lower in tumor tissues from mice treated with phenolic-rich EVOO or dEVOO compared to PEG-400-treated controls, with the most pronounced reduction observed in the OOPs group.

In vitro, only OleA treatment led to a significant increase in γH2A.X levels, whereas OOPs had no effect on γH2A.X levels. This constitutes a discrepancy with the in vivo findings which likely reflects the distinct biological contexts of the two experimental models.

### 3.7. Reduction of Oxidized Proteins by Olive Oil Phenolics in Serum of Mice with MDA-MB-231 Breast Cancer Xenografts

**Figure 7:**
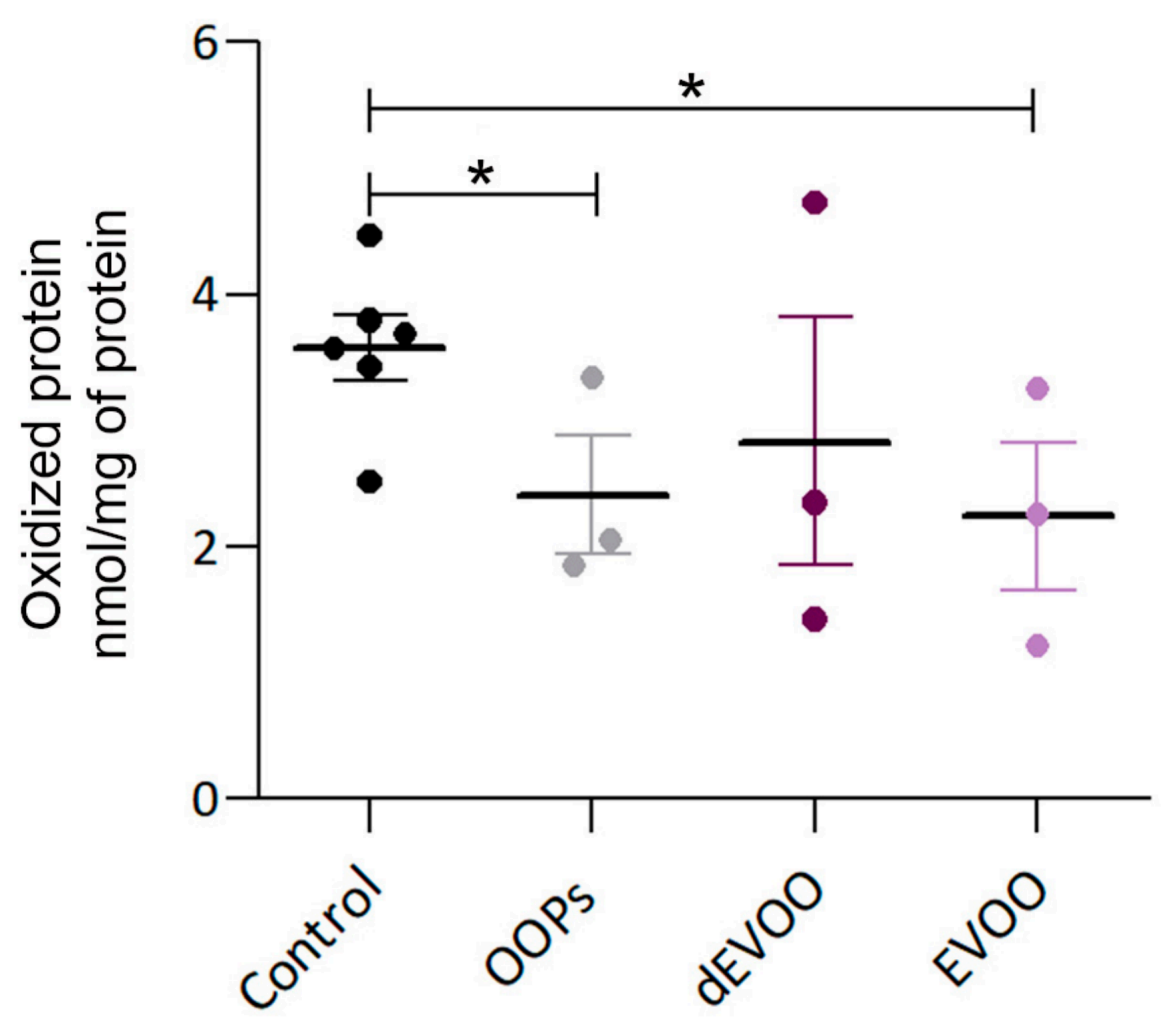
Reduction of serum oxidized proteins by OOPs or EVOO in mice bearing MDA-MB-231 breast cancer xenografts. Mice were treated for 47 days with vehicle control (PEG 400 in saline), OOPs, EVOO or dEVOO three times per week as previously described in the Materials and Methods (Section 2.6.4). Cancer cell implantation was conducted 7 days after initiation of treatment. At the study endpoint, mice were anesthetized and blood samples were collected. Serum protein carbonyl levels were measured in the serum of mice (Materials and Methods Section 2.6.4). Data are presented as mean ± SEM, with each point representing one animal (*n* = 3 mice per group). In the case of vehicle control, mice from the two prophylactic pre-treatment experiments were analyzed (*n* = 6 mice). Statistical analysis was performed using unpaired two-tailed Student’s *t*-test. *: *p* < 0.05. Abbreviations: OOPs, olive oil phenolic extract; EVOO, extra virgin olive oil; dEVOO, dephenolized extra virgin olive oil.

Oxidative stress is a major contributor to cancer progression, as it promotes macromolecular damage, including oxidative modifications of proteins that can impair cellular function and support tumor development. Protein carbonylation is a well-established and robust biomarker of oxidative protein damage [67]. In the present study, oxidized protein levels were quantified in serum samples collected from mice that received prophylactic pre-treatment with OOPs, EVOO and dEVOO, initiated seven days prior to cancer cell implantation (Figure 7).

Prophylactic pre-treatment with OOPs resulted in an approximately 33% reduction in serum oxidized protein levels compared with the vehicle control group, indicating a significant attenuation of systemic oxidative protein damage in vivo.

Notably, mice treated with dEVOO also exhibited lower serum levels of oxidized proteins than PEG-400-treated controls, indicating that olive oil constituents beyond phenolic compounds may contribute to reducing systemic protein oxidation. Consistent with this observation, treatment with EVOO produced an additional 21% reduction in serum oxidized protein levels compared to the dEVOO-treated group, highlighting the antioxidant contribution of the phenolic fraction of olive oil.

### 3.8. Effect of OOPs on MDA-MB-231 Cell Migration in Vitro

To investigate whether OOPs affect the migratory potential of TNBC cells, the IC_50_ value of the extract was first determined in MDA-MB-231 cells after 72 h exposure to the total phenolic extract (Supplementary Figure S4).

Based on the IC_50_ value determined with the MTT assay, we assessed the effect of OOPs in the ability of MDA-MB-231 breast cancer cells to migrate using the well-established wound healing assay. Cells were seeded, scratched, and cultured in reduced serum conditions (5% (*v*/*v*) to minimize proliferation rates. Cell migration was estimated as described in the Section 2.9. Migration experiments were performed using OOPs at the IC_50_ concentration (8.69 μM), as well as at subcytotoxic concentrations, corresponding to IC_50/2_ (4.35 μM) and IC_50_/_4_ (2.18 μM), over a 48 h period (Supplemtary Figure S4a,b).

**Figure 8:**
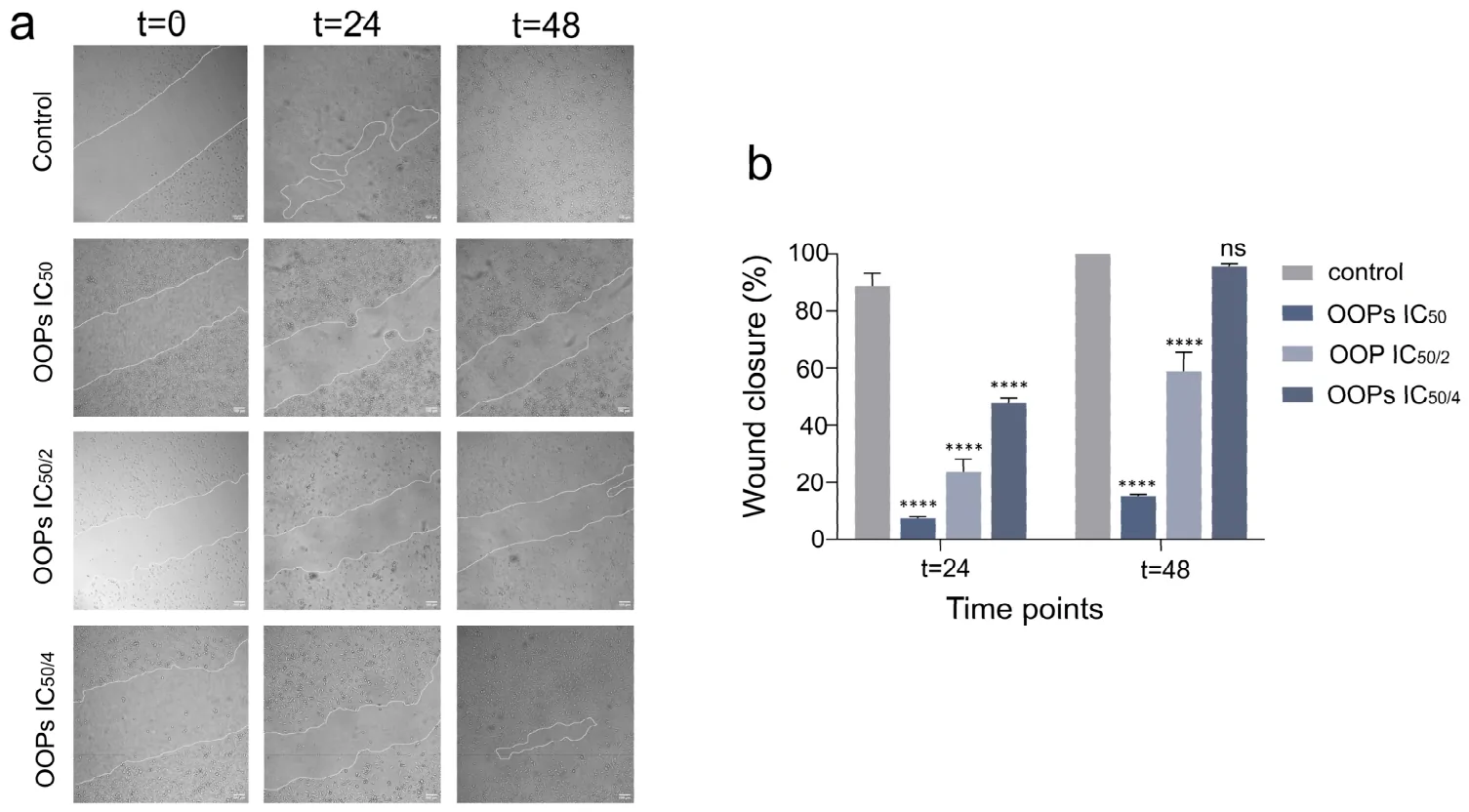
OOPs inhibit wound closure in MDA-MB-231 cells in vitro. (**a**) Representative phase-contrast images of scratched monolayers at 0, 24, and 48 h following treatment with OOPs or vehicle control. Images are from a representative experiment repeated three times in duplicate. Scale bar = 100 μm. (**b**) Quantification of wound closure over time. Wound closure (%) was calculated relative to the initial wound area at t = 0 for each well. Bars represent mean ± SEM of *n* = 6 wells (1 coverslip/well) per condition from three independent experiments. Statistics: two-way repeated-measures ANOVA (treatment × time) followed by Dunnett’s multiple comparisons test versus control within each time point. ns, not significant; **** *p* < 0.0001.

As shown in Figure 8, in vehicle-treated control samples, over time, cells progressively moved and closed the wound area which was completely healed by 48 h. In contrast, treatment with all the tested concentrations of OOPs significantly delayed wound closure in a concentration- and time-dependent manner. After 24 h, treatment with OOPs, even at the lowest concentration (IC_50/4_) not expected to affect cell proliferation (Supplementary Figure S4; [42]), reduced wound closure to approximately 48% compared with the 88% closure observed in the control samples. The IC_50_ and IC_50/2_ concentrations also significantly inhibited wound closure, resulting in values of approximately 8% and 24%, respectively.

The inhibitory effect was evident at 48 h as well, when vehicle-treated control cells exhibited complete wound closure. Cells treated with OOPs at the IC_50_ concentration showed minimal wound closure, reaching approximately 15%. Treatment with the IC_50/2_ concentration resulted in an intermediate response, with observed wound closure of about 59%, whereas IC_50/4_ allowed almost complete closure (96%).

These findings indicate that although OOPs may affect the proliferation of MDA-MB-231 cells in a concentration-dependent manner, the inhibitory effect in wound closure is mainly due to inhibition of cell migration since it was observed at subcytotoxic concentrations of IC_50/2_ and IC_50/4_ which have only a minimal effect on cell numbers in the applied experimental conditions.

## 4. Limitations

A limitation of the present study is the relatively small sample size used in the in vivo experiments. Although some treatments produced large effect sizes, the limited statistical power may have prevented biologically relevant differences from reaching statistical significance. Therefore, some findings should be considered exploratory and hypothesis-generating and the reported effect sizes interpreted as descriptive measures of treatment magnitude rather than definitive evidence of efficacy.

The study design was guided by the ethical principles of the 3Rs, with particular emphasis on the reduction of animal use. To maximize the information obtained from each animal, while avoiding pseudoreplication, a bilateral orthotopic implantation model was employed, enabling the collection of two tumor specimens per mouse for mechanistic analyses while maintaining the individual mouse as the experimental unit for efficacy analyses. It is possible that ROUT-based outlier exclusion applied for exclusion of oversized tumors may have influenced results, although this was applied uniformly to tumors from all experimental animals in the experiment described in Section 3.3 which constituted a large sample size. Nevertheless, further confirmation of these findings is needed in larger, adequately powered pre-clinical studies.

Moreover, the intraperitoneal dosing of purified compounds cannot be directly compared with the oral administration of OOPs or EVOO, as pharmacokinetic and tissue-exposure data were not obtained in the present study. Plasma and tumor concentrations of OLC, OLE, OleA, LigA, or their metabolites were not determined. Pharmacokinetic studies of olive oil phenolic metabolites are currently a work in progress, and some preliminary findings have been reported by Darakjian et al. 2021 [68] in collaboration with our team.

The mechanistic analyses presented in this study were intended to provide supportive evidence regarding potential biological pathways associated with the observed antitumor effects rather than to establish definitive mechanisms of action. Our findings suggest an association between CT-L proteasome activity, treatment composition and tumor burden in vivo, although further studies are required to determine causality. Similarly, the differential effects of OOPs on γH2AX levels in vivo and in vitro likely reflect the distinct biological contexts of the two models rather than contradictory findings. Nevertheless, the divergent γH2AX response should be interpreted cautiously and further studies investigating the temporal regulation of γH2AX signaling and the contribution of the tumor microenvironment are needed to clarify the underlying mechanisms.

## 5. Discussion

### 5.1. Dietary Olive Oil Phenolics Suppress Tumor Progression in a Triple-Negative Breast Cancer Model

This study suggests that olive oil phenolic compounds exert antitumor activity in vivo in a TNBC xenograft model and provides preliminary support for the hypothesis that these compounds may act through multiple complementary mechanisms relevant to tumor suppression. By directly comparing isolated secoiridoids, a total olive oil phenolic extract, as well as phenolic-rich EVOO within the same experimental framework, this work extends previous studies focused on individual compounds and offers additional support for the potential of olive oil phenolics as both prophylactic and adjuvant agents. The findings presented should, however, be interpreted with caution because of the limited number of experimental animals, which reduces statistical power as mentioned in Section 4 above.

The MDA-MB-231/CB17-SCID xenograft model used in this study is a well-established and widely validated model for investigating aggressive TNBC biology and therapeutic responses. CB17-SCID (cr-Prkdcscid/scid/Rj) mice provide a permissive host for human tumor engraftment due to the absence of functional T and B lymphocytes while retaining many aspects of the stromal microenvironment. Numerous studies have successfully employed CB17-SCID mice to investigate TNBC progression, metastatic dissemination, and treatment efficacy, demonstrating the robustness and reproducibility of this model [57].

Among the isolated secoiridoids administered intraperitoneally, OLC produced the greatest reduction in tumor weight, although both OleA and the OLC-OleA combination also markedly suppressed tumor burden. Combination of OLC with OleA with each compound administered at half of the monotherapy dose did not surpass the OLC monotherapy effect. Nevertheless, it retained antitumor activity despite the dose reduction and produced a numerically greater reduction in tumor burden than OleA monotherapy. The inability to produce a stronger synergistic effect in vivo may reflect differences in bioavailability, tissue distribution, metabolic stability, or tumor microenvironment interactions following systemic administration [68,69,70,71,72].

The tumor reduction observed following oral administration of OOPs, despite the lower doses of individual secoiridoids, raises the possibility that additional phenolic constituents present in the extract contribute to the overall antitumor response. Of particular interest, the antitumor effect observed with orally administered OOPs appeared numerically greater than that observed with the intraperitoneally administered OLC-OleA combination, despite the lower total secoiridoid dose in the extract-based formulation. Although this observation is consistent with the hypothesis that the activity of OOPs may reflect the combined potentially additive or synergistic actions of multiple secoiridoides, including OLC, OLE, OleA and LigA as previously demonstrated in vitro [41], this interpretation should be made with caution. The two treatment regimens differed in administration route, formulation, and control conditions, all of which may influence absorption, metabolism, bioavailability, and systemic exposure. In particular, intraperitoneal administration bypasses gastrointestinal absorption but does not necessarily avoid first-pass hepatic metabolism, whereas orally administered phenolics undergo gastrointestinal absorption followed by extensive intestinal and hepatic metabolism before reaching the systemic circulation. These pharmacokinetic differences may substantially influence the circulating metabolite profile and biological activity of the compounds. Consequently, the present study does not permit direct comparison of the efficacy of the two treatment regimens or determination of the relative contribution of individual secoiridoids to the observed antitumor effects.

The timing study highlighted that treatment initiation markedly influences the antitumor efficacy of OOPs. The greatest inhibition of tumor growth was observed with prophylactic pre-treatment administration, whereas delayed treatment after tumor cell implantation led to a progressive reduction in antitumor efficacy. This observation supports that OOPs may be particularly effective during early stages of tumor establishment, when modulation of oxidative stress and other pathways may influence the tumor microenvironment. The reduced efficacy observed with delayed treatment further indicates that olive oil phenolics may primarily limit tumor initiation and early progression rather than reverse established disease, at least at the studied doses. Our findings are consistent with previous reports demonstrating enhanced antitumor efficacy of OLC in breast cancer xenograft models when administered during early stages of tumor progression. For instance, Akl et al., 2014 [24] reported inhibition of MDA-MB-231 tumor growth following OLC treatment, which was associated with reduced expression of c-Met, Ki67, and CD31. Subsequent studies [26,29,31,39] further confirmed the antitumor efficacy of OLC across hormone-responsive, HER-2 positive and TNBC models. The present study extends these findings by directly comparing isolated secoiridoids with a total phenolic extract and phenolic-rich EVOO, while highlighting the importance of treatment timing as a determinant of antitumor efficacy. The primary objective of including these experimental groups was not to establish equivalent pharmacological potency among formulations but rather to investigate whether olive oil phenolics exert antitumor activity through different administration routes and whether phenolic-rich extracts or whole EVOO can reproduce the antitumor effects observed with isolated secoiridoids in a more nutritionally relevant context. Although phenolic-rich EVOO did not achieve statistical significance in reducing tumor weight, the observed reduction and moderate effect size indicate a biological trend toward antitumor activity and suggest potential protective effects during tumor development. Together with the findings obtained with OOPs, these results support the concept that dietary delivery of olive oil phenolics may contribute to tumor suppression, although further studies are required to validate this hypothesis. Importantly, no signs of toxicity or significant changes in body weight were observed in any treated animals, supporting the tolerability of these interventions.

### 5.2. Olive Oil Phenolics Modulate Proteasome Activity, Oxidative Stress, DNA-Damage-Related Signaling and Cell Migration

A central finding of the present study was that prophylactic administration of OOPs established a tumor-suppressive environment characterized by coordinated modulation of proteostasis, oxidative stress, DNA damage signaling and tumor cell migration. These findings suggest that the antitumor activity of olive oil phenolics is mediated through multiple complementary mechanisms that are particularly effective during the early stages of tumor establishment.

Proteostasis disruption is a hallmark of aging and cancer, and cancer cells rely on elevated proteasome activity to sustain rapid protein turnover and adapt to oxidative and proteotoxic stress [73,74,75,76].

Consistent with this concept, tumors from OOP-treated mice exhibited significantly reduced CT-L proteasome activity compared with dEVOO-treated tumors, providing direct in vivo evidence that olive oil phenolics contribute to proteasome modulation within the tumor microenvironment. In contrast, proteasome activity remained higher in EVOO- and dEVOO-treated tumors than in OOP-treated tumors, suggesting that olive-oil-mediated regulation of proteasome function involves interactions between phenolic and non-phenolic constituents. Indeed, EVOO contains several non-phenolic bioactive compounds, including triterpenes, capable of modulating redox-sensitive pathways such as Nrf2 and FOXO signaling, both closely linked to proteasome regulation [73,77,78]. These findings underscore the biochemical complexity of olive oil and highlight the need for fractionation-based studies to define the relative contribution of individual bioactive fractions. To our knowledge, this is the first study to quantitatively evaluate the effects of olive oil phenolics on overall proteasome enzymatic activity in a tumor model.

Beyond proteasome modulation, OOPs also reduced systemic oxidative stress. Protein carbonylation is a stable biomarker of oxidative protein damage in vivo [79] and has been associated with cancer progression and chronic inflammation [67]. Herein, prophylactic administration of the total OOP extract significantly reduced circulating carbonylated/oxidized serum proteins, further supporting its systemic antioxidant activity. Interestingly, dEVOO also reduced oxidized protein levels compared with vehicle-treated animals, whereas EVOO produced a greater reduction, suggesting that both phenolic and non-phenolic olive oil constituents contribute to systemic redox regulation. Improved redox homeostasis may, in turn, reduce oxidative damage to host tissues and create conditions that are less permissive for tumor progression.

Consistent with the reduction in systemic oxidative stress, OOP administration was also associated with reduced γH2A.X levels in xenograft tumors. Since γH2A.X is a sensitive marker of DNA double-strand breaks (DSBs), these findings suggest reduced activation of DNA damage signaling and decreased genotoxic stress in vivo [80]. Previous in vitro studies have similarly demonstrated that olive oil extracts, hydroxytyrosol, and related olive phenolics reduce oxidative DNA damage in various cell types [80,81,82,83,84].

Lower γH2A.X levels are consistent with reduced DNA damage signaling, fewer replication-associated lesions, and decreased chromosomal instability, all of which are linked to suppression of tumor initiation and progression [85]. The observed reduction in γH2A.X levels may reflect modulation of oxidative stress pathways by OOPs, resulting in lower activation of DNA damage signaling [86]. However, because γH2A.X is an indirect marker, further studies are needed to determine whether these changes correspond to reduced DSB formation, altered DNA repair dynamics, or both.

The discrepancy between the in vivo reduction and the lack of an in vitro effect of OOPs on γH2A.X levels observed in this study likely reflects the distinct biological contexts of the two experimental models. In vivo, γH2A.X levels in xenograft tumors represent the cumulative outcome of prolonged, repeated low-dose exposure to metabolized phenolic compounds together with changes in oxidative stress, systemic metabolism, tumor progression and interactions within the tumor microenvironment, all of which may influence DNA damage signaling. In contrast, cultured MDA-MB-231 cells were exposed acutely (24 h) to parent compounds or extracts at IC_50_ concentrations, reflecting only the direct cellular response. Furthermore, the OOP extract contains multiple phenolics that may exert complementary or opposing biological effects, while their metabolism and tissue distribution in vivo cannot be reproduced under cell culture conditions. The concentration-dependent antioxidant and pro-oxidant properties of olive phenolics [87,88] may also contribute to the differential γH2A.X responses observed. Therefore, the in vivo and in vitro models address complementary rather than identical biological processes and the reduction in γH2A.X levels observed following prophylactic OOP treatment in vivo should not be interpreted as directly corresponding to the in vitro findings. Nevertheless, this divergence represents a limitation of the present study and highlights the need for further investigations to elucidate the mechanisms underlying the context-dependent regulation of γH2A.X. Moreover, the biological activity observed in the dEVOO group indicates that non-phenolic constituents of olive oil may also contribute to the modulation of γH2AX levels and oxidative protein damage and the effects observed cannot be attributed exclusively to the phenolic fraction.

Beyond serving as a DNA damage marker, γH2A.X is involved in apoptosis and cell survival signaling. Persistent DSBs activate ATM/Chk2 or p53-mediated apoptosis, whereas lower DSB levels help maintain controlled apoptotic thresholds [89]. Reduced γH2A.X levels may therefore support cellular homeostasis, by preserving healthy cells while enabling regulated elimination of damaged ones. This environment may also contribute to reduced oxidative stress within the tumor microenvironment.

Overall, early OOP treatment appears to establish a tumor-suppressive environment characterized by reduced genotoxic stress, improved genome maintenance, stabilized redox balance and modulation of apoptosis. These findings highlight the importance of treatment timing in maximizing the genoprotective and tumor-suppressive potential of olive oil phenolics.

Consistent with the observations described above, OOPs inhibited migration of MDA-MB-231 cells in vitro, even at subcytotoxic concentrations, suggesting that the complex mixture of phenolics may contribute to the inhibition of tumor cell migration. This inhibition exceeded that previously reported for individual olive oil phenolics, such as OLC, OleA or hydroxytyrosol [90,91,92], suggesting a potentially additive or synergistic activity within the complex phenolic mixture. Although the results obtained with the EC_50_ concentration of OOPs may reflect both reduction of cell numbers and inhibition of migration, the results with the EC_50/2_ and EC_50/4_ concentrations of OOPs reflect mainly inhibition in cell migration. Sub-EC_50_ concentrations of OOPs affect minimally cell numbers at 24–72 h (Supplementary Figure S4 and [42]). Therefore, the observed results in the wound healing assay for the EC_50/2_ and EC_50/4_ OOP concentrations correspond mainly to effects in cell migration. Translational interpretation of these findings requires careful consideration of the relationship between in vitro and in vivo exposure. Animal studies commonly administer olive phenolic extracts in the range of ~10–125 mg/kg/day, with 25 mg/kg/day frequently used, whereas in vitro experiments often rely on higher concentrations of pure isolated compounds (10–100 μM) [22,93,94,95,96]. Extract-based studies generally report effects at concentrations of approximately 25–100 μg/mL, corresponding to similar micromolar ranges depending on the phenolic composition [95]. Notably, some inhibitory effects have also been observed at lower, more physiologically relevant concentrations, such as around 10 μM oleuropein or 1 μM hydroxytyrosol in hepatocellular carcinoma models [97]. In humans, plasma levels of free hydroxytyrosol following standard EVOO consumption are very low (≤0.05 μM); however, phenolic-enriched oils can increase circulating metabolite levels, such as that of hydroxytyrosol sulfate, to 1–4 μM. Importantly, most in vitro studies examine the effects of free phenolics, whereas in vivo phenolics predominantly circulate as metabolites, with potentially distinct bioactivities. Collectively, these considerations underscore the challenges in extrapolating in vitro findings to human exposure and emphasize the challenge to account for both concentration and metabolite form when evaluating antimetastatic potential of olive oil phenolics.

## 6. Conclusions

This study provides in vivo observations suggesting the anticancer potential of OOPs in a human TNBC xenograft mouse model and identifies treatment timing as a critical determinant of efficacy with prophylactic pre-treatment producing the greatest effect in suppressing tumorigenesis. By comparing isolated olive oil secoiridoids, a total olive oil phenolic extract (OOPs), and phenolic-rich extra virgin olive oil (EVOO), this work indicates that complex phenolic mixtures may exert greater biological activity than individual compounds, likely through complementary or synergistic interactions among phenolic constituents and their circulating metabolites. The integration of in vivo antitumor efficacy with mechanistic analyses including proteasome activity, γH2AX signaling, oxidative protein damage and cell migration supports a multimodal mechanism of action. This multitarget activity of OOPs is consistent with emerging strategies in breast cancer research, where agents acting on multiple pathways have shown promise in overcoming resistance mechanisms [98].

Taken together, these findings highlight the potential of olive oil phenolics, particularly complex phenolic mixtures, as prophylactic agents in TNCB and highlight the importance of further investigation into the contribution of bioavailability, metabolism and phenolic interactions to their anticancer activity.

## Data Availability

The original contributions presented in this study are included in the article/Supplementary Materials. Further inquiries can be directed to the corresponding authors. Any additional raw data supporting the conclusions of this article may be made available by the authors on request.

## Supplementary Materials

The following supporting information can be downloaded at: https://www.mdpi.com/article/10.3390/nu18172756/s1, Figure S1: ^1^H NMR spectrum (400 MHz, CDCl_3_) of the enriched OOP extract used for in vivo administration; Figure S2: ^1^H NMR spectrum (400 MHz, CDCl_3_) of purified OLC isolated from olive oil phenolic extracts. Expanded spectral regions highlight characteristic resonances used for compound identification; Figure S3: ^1^H NMR spectrum (400 MHz, CDCl_3_) of OleA isolated from olive oil phenolic extracts. Expanded spectral regions highlight characteristic resonances used for compound identification. OleA is present as two isomeric forms in solution. The aldehydic resonance at δ 9.54 ppm corresponds to the major isomer (isomer I), while δ 9.61 ppm corresponds to the second isomer (isomer II). Signal assignments are indicated on the structure and spectrum; Figure S4: OOP cytotoxicity-based dose selection in MDA-MB-231 cells. (a) Dose–response curve showing cell viability after 72 h exposure to increasing concentrations (2–200 μM) of OOPs, assessed by MTT assay. Data represent mean ± SEM from three independent experiments, each performed in triplicate. (b) Bar graph of IC_50_ value (mean ± SD) for OOP, derived from the same experiments. Figure S5:Representative confocal immunofluorescence images from wound healing assays of MDA-MB-231 cells treated with OOPs at the IC_50_ concentration for 12 h. F-actin was stained with CF^®^633-phalloidin (magenta) and nuclei with Hoechst 33342 (cyan). Images were acquired at the wound margins to visualize cell migration following treatment. Scale bars: 100 μm. Table S1 Quantitative 1H NMR characterization of the OOP formulation used for in vivo dosing. Composition was determined by qNMR using syringaldehyde as internal standard, following the protocol described by Karkoula et al. 2014 [56]. Values represent the percentage (*w*/*w*) of each quantified phenolic compound in the OOP extract; Table S2: Sensitivity analysis of tumor burden according to ROUT-based outlier handling. Total tumor burden was calculated at the mouse level as the sum of the bilateral tumor weights, with the individual mouse considered the experimental unit. The analysis with ROUT excluded the entire mouse when one of its two tumors had been identified as an outlier by the ROUT method (Q = 1%). The analysis without ROUT included all mice and all tumor measurements, with no outlier exclusions. Treatment groups were compared with the vehicle control using one-way ANOVA. Exact adjusted *p* values, mean differences, and 95% confidence intervals (CIs) are reported.
