# Supplementary material for: Time-Dependent Multimechanistic Antitumor Effects of Olive Oil Phenolics in a Triple-Negative Breast Cancer Mouse Model
Source: Nutrients. 2026 Aug 23;18(17):2756. doi: 10.3390/nu18172756 (PMC13567287; doi:10.3390/nu18172756)
Supplement: Supplementary file 1 [file nutrients-18-02756-s001.zip › nutrients-4422513 - Supplementary.pdf]

## Supplementary Material

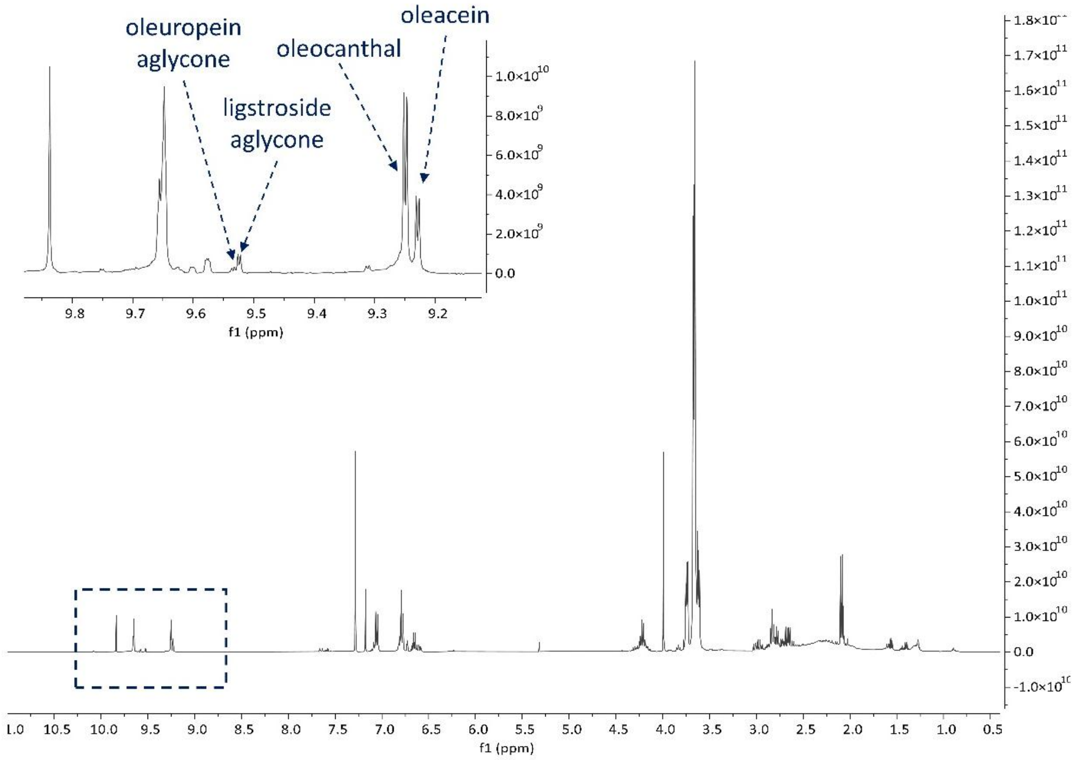


**Figure S 1** $^1$H NMR spectrum (400 MHz, $CDCl_3$) of the enriched OOPs extract used for in vivo administration. The extract consisted primarily of oleocanthal (OLC), oleacein (OLE), oleuropein aglycone (OleA), and ligstroside aglycone (LigA), together with minor amounts of tyrosol and hydroxytyrosol. Quantitative analysis was performed as described in Materials and Method

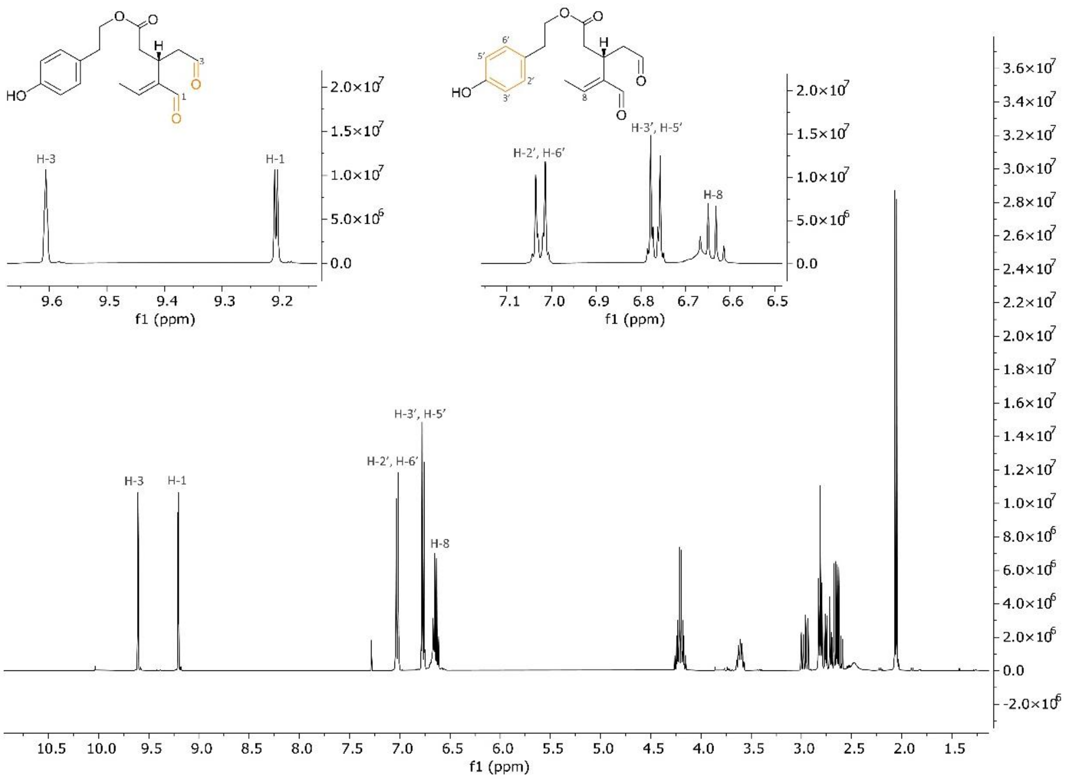


**Figure S2** $^{1}H$ NMR spectrum (400 MHz, $CDCl_3$) of purified OLC isolated from olive oil phenolic extracts by reverse-phase flash chromatography. Compound identity and purity (≥97 %) were confirmed by NMR analysis prior to biological evaluation. $^{1}H$ NMR spectrum (400 MHz, $CDCl_3$) of purified OLC isolated from olive oil phenolic extracts. Expanded spectral regions highlight characteristic resonances used for compound identification.

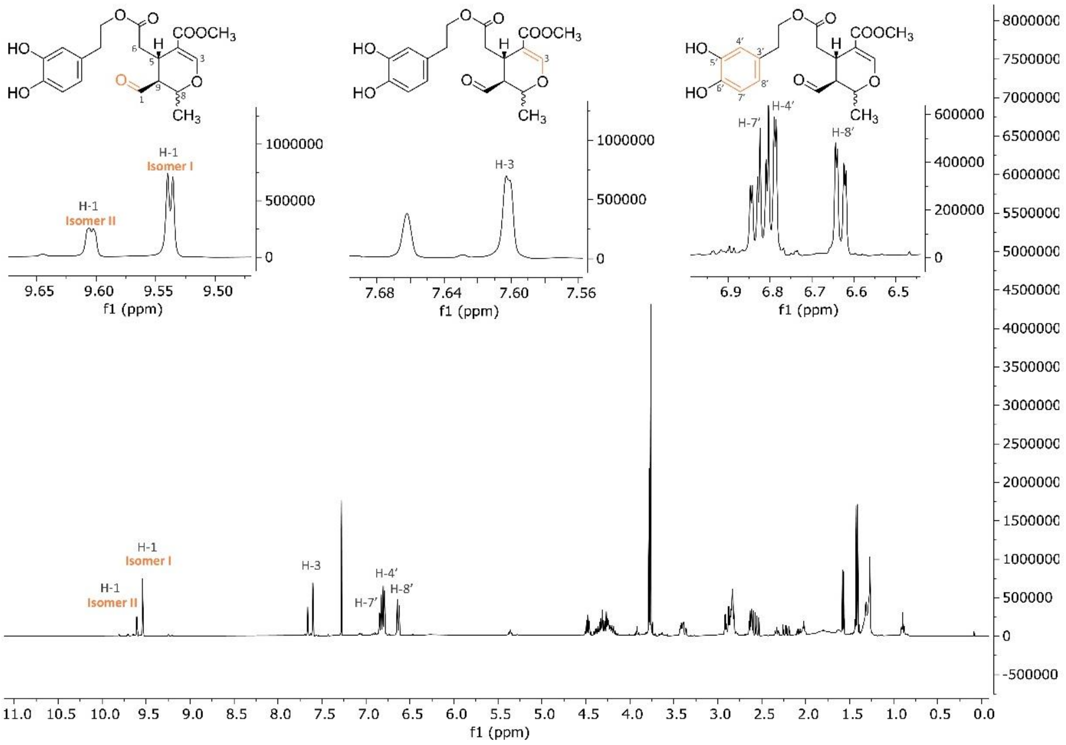


**Figure S3** $^{1}$H NMR spectrum (400 MHz, $CDCl_3$) of OleA isolated from olive oil phenolic extracts. Expanded spectral regions highlight characteristic resonances used for compound identification. OleA is present as two isomeric forms in solution. The aldehydic resonance at δ 9.54 ppm corresponds to the major isomer (isomer I), while δ 9.61 ppm corresponds to the second isomer (isomer II). Signal assignments are indicated on the structure and spectrum.

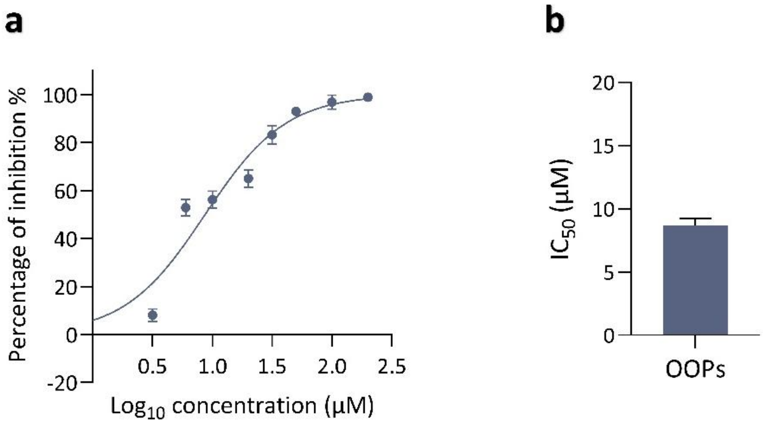


**Figure S4** OOPs cytotoxicity-based dose selection in MDA-MB-231 cells**.** (a) Dose–response curve showing cell viability after 72h exposure to increasing concentrations (2-200 μM) of OOPs, assessed by MTT assay. Data represent mean ± SEM from three independent experiments, each performed in triplicate. (b) Bar graph of $IC_{50}$ value (mean ± SEM) for OOPs, derived from the same experiments.

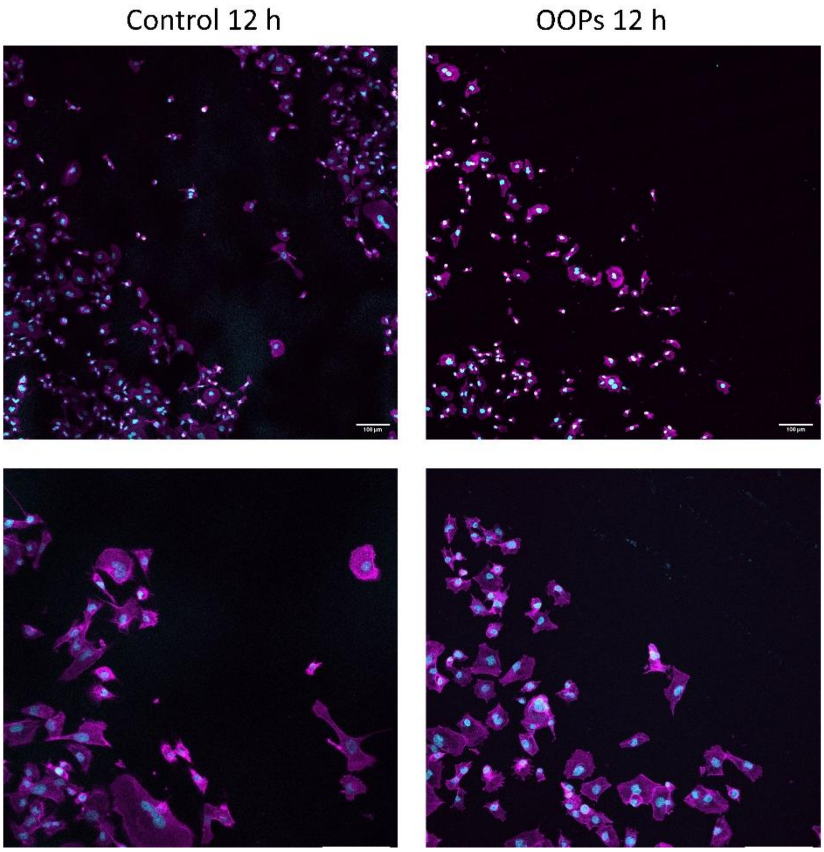


**Figure S5** Representative confocal immunofluorescence images from wound-healing assays of MDA-MB-231 cells treated with OOPs at the $IC_{50}$ concentration for 12 h. F-actin was stained with CF®633-phalloidin (magenta) and nuclei with Hoechst 33342 (cyan). Images were acquired at the wound margins to visualize cell migration following treatment. Scale bars: 100 μm.

**Table S 1** Quantitative $^{1}H$ NMR characterization of the OOPs formulation used for *in vivo* dosing. Composition was determined by qNMR using syringaldehyde as internal standard, following the protocol described by Karkoula et al. 2014. Values represent the percentage (w/w) of each quantified phenolic compound in the OOPs extract, as mean ± SD from n = 3 determinations.

| **Compound** | **Integration** | **Quantification signal (δ, ppm)** | **Content in extract (%, w/w)** | **Ratio to OLC** |
|---|---|---|---|---|
| Oleocanthal | 3.24 | 9.23 | 21.60 ± 0.05 | 1.00 |
| Oleacein | 1.48 | 9.19 | 10.40 ± 0.03 | 0.48 |
| Oleuropein aglycone | 0.26 | 9.51 | 2.16 ± 0.2 | 0.10 |
| Ligstroside aglycone | 0.65 | 9.49 | 5.12 ± 0.3 | 0.24 |
| Tyrosol | 0.12 | 7.11 | 0.40 ± 0.02 | 0.02 |
| Hydroxytyrosol | 0.11 | 6.57 | 0.40 ± 0.03 | 0.02 |

Internal standard: syringaldehyde. *For quantitative analysis of hydroxytyrosol, an aliquot of the OOPs extract was dissolved in $CD_3OD$ prior to NMR analysis. Integrations and quantification performed as described in Section 2.2.

**Table S2: Sensitivity analysis of tumor burden according to ROUT-based outlier handling.** Total tumor burden was calculated at the mouse level, with the individual mouse considered the experimental unit. The analysis with ROUT excluded the individual tumor measurements identified as outliers by the ROUT method (Q = 1%). For mice with an identified outlier, the remaining bilateral tumor measurement was used to calculate the mouse-level tumor burden. The analysis without ROUT retained all mice and both bilateral tumor measurements, which were summed to calculate total tumor burden for each mouse, with no outlier exclusions. Treatment groups were compared with the vehicle control using one-way ANOVA. Exact adjusted P values, mean differences, and 95% confidence intervals (CI) are reported.

| Experimental group | Analysis | *P* value | 95% CI of diff | Mean diff. |
|---|---|---|---|---|
| OOPs (-7d) | With ROUT | 0.0008 | 0.07154 to 0.2838 | 0.1777 |
| | Without ROUT | 0.9426 | -0.2860 to 0.4142 | 0.06411 |
| OOPs (+5d) | With ROUT | 0.0439 | 0.003152 to 0.2747 | 0.1389 |
| | Without ROUT | 0.9943 | 0.3805 to 0.4480 | 0.03375 |
| OOPs (+20d) | With ROUT | 0.2559 | -0.04435 to 0.2272 | 0.09143 |
| | Without ROUT | 0.7656 | -0.5480 to 0.2805 | -0.1338 |

**Table note:** "With ROUT" indicates exclusion of one bilateral tumor identified as an outlier (Q = 1%), with the remaining tumor measurement used to calculate the mouse-level tumor burden for that mouse. "Without ROUT" indicates that both bilateral tumor measurements were retained and summed to calculate total tumor burden for each mouse. The individual mouse was the experimental unit in both analyses.
